# Liquid biopsies and minimal residual disease in lymphoid malignancies

**DOI:** 10.3389/fonc.2023.1173701

**Published:** 2023-05-09

**Authors:** Maroun Bou Zerdan, Joseph Kassab, Ludovic Saba, Elio Haroun, Morgan Bou Zerdan, Sabine Allam, Lewis Nasr, Walid Macaron, Mahinbanu Mammadli, Sarah Abou Moussa, Chakra P. Chaulagain

**Affiliations:** ^1^ Department of Internal Medicine, State University of New York (SUNY) Upstate Medical University, Syracuse, NY, United States; ^2^ Cleveland Clinic, Research Institute, Cleveland, OH, United States; ^3^ Department of Hematology-Oncology, Myeloma and Amyloidosis Program, Maroone Cancer Center, Cleveland Clinic Florida, Weston, FL, United States; ^4^ Department of Medicine, State University of New York (SUNY) Upstate Medical University, New York, NY, United States; ^5^ Faculty of Medicine, American University of Beirut, Beirut, Lebanon; ^6^ Department of Medicine and Medical Sciences, University of Balamand, Balamand, Lebanon; ^7^ University of Texas MD Anderson Cancer Center, Texas, TX, United States; ^8^ Faculty of Medicine, Université Paris-Saclay, Paris, France

**Keywords:** lymphoid malignancies, liquid biopsy, minimal residual disease, multiple myeloma, oncology

## Abstract

Minimal residual disease (MRD) assessment using peripheral blood instead of bone marrow aspirate/biopsy specimen or the biopsy of the cancerous infiltrated by lymphoid malignancies is an emerging technique with enormous interest of research and technological innovation at the current time. In some lymphoid malignancies (particularly ALL), Studies have shown that MRD monitoring of the peripheral blood may be an adequate alternative to frequent BM aspirations. However, additional studies investigating the biology of liquid biopsies in ALL and its potential as an MRD marker in larger patient cohorts in treatment protocols are warranted. Despite the promising data, there are still limitations in liquid biopsies in lymphoid malignancies, such as standardization of the sample collection and processing, determination of timing and duration for liquid biopsy analysis, and definition of the biological characteristics and specificity of the techniques evaluated such as flow cytometry, molecular techniques, and next generation sequencies. The use of liquid biopsy for detection of minimal residual disease in T-cell lymphoma is still experimental but it has made significant progress in multiple myeloma for example. Recent attempt to use artificial intelligence may help simplify the algorithm for testing and may help avoid inter-observer variation and operator dependency in these highly technically demanding testing process.

## 1 Liquid biopsies

### 1.1 Liquid biopsy components

#### 1.1.1 Circulating tumor cells

Circulating tumor cells (CTCs) are cancerous cells that break away from the primary tumor and enter the bloodstream, leading to metastasis. The detection of CTCs in clinical samples using a liquid biopsy can be an effective tool for cancer diagnosis and prognosis (1). However, CTCs are present in low abundance in most patients and have a short half-life of a few hours, making them difficult to detect. Various techniques have been defined to isolate and analyze CTCs with their detection usually dependent on molecular markers like the epithelial cell adhesion molecule - the most widely used (2, 3). Though still experimental, CTC detection is now used as a surrogate biomarker in numerous solid cancers (breast, prostate, lung, and liver cancers). The analysis CTCs is not commonly done in hematological malignancies, but it is still possible in certain conditions such as acute leukemias, myelodysplastic syndromes (MDSs), myeloproliferative neoplasms (MPNs), multiple myelomas (MM), and lymphomas (4–6).

#### 1.1.2 Cell-free DNA

It has been known for some time that circulating cell-free DNA (cfDNA) can be found in human body fluids, including plasma. In healthy individuals, the majority of cfDNA molecules in plasma originate from the hematopoietic system. However, in certain clinical situations, such as pregnancy, organ transplantation, and cancer, it has been observed that the related or affected tissues may release additional DNA into the plasma pool (7, 8). In oncological patients, apoptosis, necrosis, or both, are the main sources of circulating cell-free DNA (cfDNA) (in this case called circulating tumor DNA - ctDNA). Apoptosis is a process of programmed cell death characterized by the trimming of chromosomes into nucleosomes (consisting of 180 base pairs) by enzymes called DNases, which are then released into the bloodstream. These nucleosomes circulate for a period of one to three hours before being removed by phagocytic cells and other cells. Necrosis, on the other hand, occurs when cells are damaged or stressed to the point of uncontrolled death, leading to the random and incomplete digestion of DNA (9–11). It has been suggested that the fragmentation profile of circulating cfDNA may reveal information about the tissue origin in cancer patients (11). Additionally, DNA methylation patterns are known to be tissue-specific, and thus, their analysis may allow for the determination of the origin of cf-DNA fragments. Plasma cfDNA methylation profiling has also been demonstrated to be a highly accurate approach for the diagnostic evaluation of several types of malignancies (12, 13). Furthermore, the sequence analysis of tumor-associated cfDNA may also be utilized for the identification of specific mutations, such as *TP53, KRAS, BRAF*, and *HER2*, which may be useful for guiding therapeutic decisions (14, 15). Liquid biopsies, by the quantification of cfDNA using real-time quantitative digital polymerase chain reaction (qRT-dPCR) and next-generation sequencing (NGS), can be used for all types of lymphomas, because the plasma regularly contains low levels of detectable lymphoma-derived cfDNA, as well as in myeloid pathology, MDS, MPN, and AML, and/or other lymphoid malignancies, such as acute lymphoblastic leukemia (ALL) and multiple myeloma (MM) (16, 17).

#### 1.1.3 Tumor-educated platelets

Numerous studies demonstrated that platelets could influence various aspects of cancer progression, particularly tumor cell metastasis. In cancer patients, platelets often display distinct RNA and protein profiles, despite showing no significant changes in their hemostatic activity. This phenotypically distinct population of platelets, known as tumor-educated platelets (TEP), has recently received considerable attention due to its potential detection and analysis using liquid biopsy (18, 19).

#### 1.1.4 Exosomes

Exosomes are small, extracellular vesicles that are secreted by multivesicular bodies within live cells through exocytosis. They range in size from 30 to 150 nm and can facilitate communication between cells due to their composition, which includes nucleic acids, proteins, and lipids (20). Tumor-derived exosomes (TDEs) are closely related to tumor progression, metastasis, and immune evasion. Thus, TDEs analyzed through their RNA content, may hold great promise for early cancer detection, prognosis, and management assessment (21, 22).

#### 1.1.5 Micro-RNA

MicroRNAs (miRNAs) are small, non-coding RNA molecules that are about 20 nucleotides long and play a role in regulating posttranscriptional gene expression. In recent years, miRNAs have received increasing attention due to their involvement in various aspects of cancer progression, such as cell growth, differentiation, proliferation, angiogenesis, and apoptosis (23, 24). The high concentration of miRNAs in exosomes has led to a growing interest in the use of exosomal miRNAs as cancer biomarkers for purposes of diagnosis, prognosis, and therapeutics (25, 26).

## 2 Minimal residual disease assessment using liquid biopsy

In the setting of hematological malignancies, minimal residual disease (MRD) is defined as the presence of residual leukemic cells not detected by conventional cytomorphological methods, and it is a powerful parameter to guide clinical management. For instance, MRD is accepted as the strongest independent prognostic factor in ALL (27). MRD can be performed on bone marrow aspirate specimens or blood- liquid biopsy through the detection of ctDNA. The preferred approach for detecting MRD varies according to immunophenotype and molecular features of the cancerous cells and available resources. Methods of analysis can vary but they mainly include the following. First is multicolor flow cytometry which is a rapid method that identifies leukemic blasts based on their aberrant immunophenotype. It is less sensitive than molecular techniques and may produce false-negative results if the immunophenotype changes (28). Second is through polymerase chain reaction (PCR) which can quantify MRD by detecting blasts based on immunoglobulin (Ig) or T-cell receptor (TCR) gene rearrangements or leukemia-specific chromosomal rearrangements. It is more sensitive than flow cytometry, but requires a sample obtained at the time of diagnosis and may produce false positives due to sample contamination (29, 30). The third method is reverse transcriptase PCR(RT-PCR) which can identify leukemia-specific fusion mRNA, such as the BCR : ABL1 rearrangement associated with t (9, 22). It is very sensitive and stable throughout the disease course but is only applicable for a minority of patients and the mRNA target may be more easily degraded during sample handling/transport compared to DNA (31, 32). Last, next-generation sequencing (NGS) uses multiplex PCR to amplify Ig or T-cell receptor (TCR) gene rearrangements and then sequences the DNA with high depth (coverage). It is the most sensitive method for detecting MRD and enables monitoring of MRD and identification of clonal evolution during therapy (32).

Numerous studies have demonstrated that the positive predictive value of ctDNA’s detection, is satisfactory since the presence of MRD, which can be detected through its presence, is linked to a poorer disease-free survival rate. However, many limitations maintain the negative predictive of this technique at a low level. They include detection methods that are not sensitive enough to detect ctDNA at very low levels, improper timing of post-treatment sampling, and the possibility of new cell clones emerging that are not detected by the chosen technique. In addition, proper sample processing logistics are critical for the adequate detection of cfDNA and to prevent the breakdown of WBCs and contamination with genomic DNA. It is ideal to process the samples quickly (preferably within four hours) or to collect them in tubes containing a cell stabilizing agent (33–35). **Table 1** summarizes the available MRD detection techniques in different lymphoid malignancies.

**Table 1:** Assessment of MRD in lymphoid malignancies.

Method	ALL	DLBCL	FL	HL	CLL	MM
**Imaging**	–	PET/CT scan	PET/CT scan	PET/CT scan	Lymphoid nodes, spleen, and liver evaluation by CT	PET/CT scan
**Histology**	BM biopsy	BM biopsy (facultative)	BM biopsy (facultative)	Only if BM involvement at the time of diagnosis	BM biopsy	BM biopsy
**MFC**	Yes	–	–	–	Yes	Yes
**Molecular**	RQ-PCR	–	–	–	RQ-PCR	–
**NGS**	NGS Igs-TCR	NGS liquid biopsy, investigational use	NGS liquid biopsy, investigational use	Investigational use	NGS Igs	NGS Igs
**Timing**	After completion of initial induction.	Post-third cycle every 3 months	Post-third cycle every 3–6 months	Post-third cycle. Additional time points should be guided in accordance with the regimen used	Post-third cycle every 3–6 months	Post-third cycle. every 6 months
**References**	(36)	(37)	(37)	(38)	(39)	(40)

ALL, acute lymphoblastic leukemia; DLBCL, diffuse large B-cell lymphoma; FL, follicular lymphoma; HL, Hodgkin lymphoma; CLL, chronic lymphatic leukemia; MM, multiple myeloma; CT, computed tomography; PET, positron emission tomography; BM, bone marrow; MFC, multiparametric flow cytometry; RQ-PCR, real quantitative PCR; PCR, polymerase chain reaction; NGS, next-generation sequencing; MRD, minimal residual disease. -, Not present.

## 3 MRD assessment using liquid biopsy in different malignancies

### 3.1 ALL

MRD is the most important prognostic factor in ALL and can be used to shape treatment choices and overall patient management to achieve better outcomes (41). Multiparameter flow cytometry, PCR based diagnostics used in ALL with *BCR/ABL1* mutations, or tests based on next-generation sequencing (NGS) of clonal Ig and/or TCR loci can all be used to assess ALL MRD (42, 43). Compared to the other modalities, NGS is the most sensitive for ALL MRD detection in the bone marrow (BM) with a sensitivity of 10^-6^, it is approved and commercially accessible in the United States, and it is being used more frequently in clinical practice (44). However, these approaches’ requirement for recurrent invasive BM assessments is one of their limitations, especially in the pediatric population that constitutes 60% of newly diagnosed ALL cases (45). Hence, liquid biopsies would be a significant advancement to avoid these traditional intrusive tests. Yet only a handful of studies have explored the use of blood for MRD assessment in ALL. Schwartz et al. demonstrated that the elevation of total cfDNA in plasma in ALL could possibly be used as an MRD marker. They also revealed using quantitative analysis that Ig/T cell receptor (TCR) gene rearrangements showed significant concordance between plasma and peripheral blood (PB) leukocytes (46). Furthermore, a recent study showed that monitoring plasma for leukemia structural variants (SVs), found in cfDNA, with droplet digital PCR (ddPCR) may provide useful information for the tailored follow-up of ALL patients (47). In their study of Ig/TCR rearrangements in bone marrow compared to peripheral blood (PB) samples from T-ALL and precursor B-ALL, Van der Velden et al. discovered a high association between the BM and PB MRD levels of T-ALL but not of precursor B-ALL (48). Another study on pediatric ALL looked into the level of clonal Ig/TCR gene rearrangements in plasma and compared it to MRD determined by flow cytometry in BM. The authors came to the conclusion that both approaches could independently predict relapse despite the methods’ poor correlation (49). All these studies have shown that MRD monitoring of the PB appears to be an adequate alternative to frequent BM aspirations, however, additional studies investigating the biology of liquid biopsies in ALL and its potential as an MRD marker in larger patient cohorts within current treatment protocols are warranted.

### 3.2 Diffuse large B cell lymphoma

Some malignant cells express antigen receptors of B-lymphocytes (BCR). These receptors are made up of paired light and heavy immunoglobulin chains. Variable diversity joining (VDJ) rearrangements in the progenitor B-cells can result in diversity in the characteristics of molecules which lead to unique clonotypes in each lymphoma. These can be useful in anticipating relapses in the peripheral blood if the relapse comes from the diagnosis clone. This technique has some caveats as some DLBCLs have unproductive VDJ rearrangements (50, 51). However, multiple studies have shown that liquid biopsies, specifically cfDNA and ctDNA analysis, can be a practical tool in determining minimal residual disease in DLBCL (52–56).

Assessing the response to the treatment, prognosis, and early detection of relapse is a complex and sometimes inaccurate process due to the biological and clinical heterogeneity of DLCBL. Liquid biopsies have also been shown to be effective in diagnosis, evaluation of disease progression, and response to the treatment of DLBCL (54, 57–61). Liquid biopsies, such as ddPCR, have also been utilized to detect common mutations in DLBCL such as *XPO1, EF71K, EZH2 Y641*, and *MYD88 L265P* (54, 57, 62). Roschewki et al. evaluated the appropriateness and prognostic impact of NGS-based molecular ctDNA (63). Rossi et al. assessed the rapid clearance of mutations from cfDNA among the responsive patients with DLBCL treated with R-CHOP. This was demonstrated using CAncer Personalized Profiling by deep Sequencing (CAPPseq) technology (64). A high ctDNA burden before treatment has been proven to be associated with a bad prognosis and high MRD (53, 60). This concept was introduced by Kurtz and his colleagues in the evaluation of the response and determining MRD by ctDNA analysis prior to the chemotherapy (65). Pretreatment ctDNA and molecular responses were independently prognostic of outcomes in DLBCL. Furthermore, a two-log drop in ctDNA levels after two chemotherapy courses were associated with an eventual completer response and cure (63). Later Kurtz and his colleagues used phased variant enrichment and detection sequencing (PhasED-Seq), where they were able to detect phase variants for DBLCL and increase the sensitivity (66). To overcome the heterogeneity and to detect MRD, different fragmentation patterns and hypermutation patterns of ctDNA were identified; they were shown to be superior to traditional tissue biopsies in terms of prognostic information (67). In this study, the latter also accurately determined the absence of MRD in cured patients where PET falsely identified the presence of MRD (67). These studies showed that ctDNA analysis could be informative and even superior in DLBCL compared to the other methods. Another group of scientists decided that the tumor burden by ctDNA prior to treatment heavily relies on the diagnosis to treatment interval (68). Shin et al. analyzed the plasma of cfDNA of a proposed a panel of 66 genes associated with non-Hodgkin’s lymphoma. This study revealed that the level of ctDNA was decreased in patients with a response to therapy, and it increased in patients with disease progression (69). Last, monitoring the MRD with ctDNA may be helpful, following CAR-T-cell therapy (37, 70).

Exosomes or exosomal nucleic acids are another type of liquid biopsy that is shown to be informative in determining MRD and response to treatment (71–73). Furthermore, exosomes also are promising in exploring the new therapeutic targets in DLBCL. Chen and her colleagues provided evidence that Tumor-derived exosomes can increase the immune response and promote tumor growth, suggesting that targeting exosomes could be a potential target in the treatment of DLBCL (74).

Along with the promising data, there are still limitations in liquid biopsies in the context of DLBCL, such as standardization of the sample collection and processing, determination of timing and duration for liquid biopsy analysis, and definition of the biological characteristics and specificity of the ctDNA, cfDNA, and exosomes. One must keep in mind that some mutations such as *TP53* or *DNMT3A* could have their origin in clonal hematopoiesis of an indeterminate potential (CHIP) (58, 75). Providing more convincing evidence by comparing the different methods of MRD detection versus liquid biopsies is crucial. Nevertheless, there is promising evidence that, soon we will be able to use liquid biopsies in the assessment of the MRD in DLBCL and by that could diminish the invasive procedures and overtreatment by false positive MRD.

### 3.3 Mantle cell lymphoma

Mantle cell lymphoma (MCL) is a rare and aggressive type of non-Hodgkin lymphoma, with a median survival of five to six years. As MCL significantly invades both bone marrow and peripheral blood, MRD assessment has been useful in the management of MCL and has been developing over time from flow cytometry and PCR, to more recent NGS-based methods (76). In a randomized phase 2 trial of bendamustine + rituximab-based induction followed by rituximab ± lenalidomide consolidation for MCL, MRD levels have been shown to be correlated with progression-free survival (PFS) (77). In another study in patients with MCL, post-stem cell transplant (SCT) MRD tracking using IgNGS had a high negative predictive value and was able to identify the presence of ctDNA months prior to clinical recurrence (78).

Lakhotia et al. analyzed the clinical significance of ctDNA dynamics by NGS during therapy in patients with MCL treated with bortezomib + DA-EPOCH-R. Baseline ctDNA was significantly correlated with the total metabolic tumor volume (TMTV) on PET Scan, and dynamic changes that occurred very early during therapy predicted clinical outcomes. ctDNA clearance after one and two cycles of therapy were significantly associated with a longer PFS, and a trend towards better overall survival (OS) was seen. Additionally, ctDNA monitoring after induction showed that molecular relapse can precede clinical relapse (79). Similarly, another prospective phase 2 study of sequential chemoradioimmunotherapy followed by SCT showed that post-treatment NGS–based ctDNA monitoring identified early molecular relapse. In patients who relapsed, 67% had MRD positivity at least 3 months prior, and 72% of patients who did not relapse had undetectable ctDNA (80).

### 3.4 Follicular cell lymphoma

Several studies have shown that quantification of cfDNA and ctDNA in follicular cell lymphoma (FL) at diagnosis represents a potential powerful prognostic biomarker. Using NGS to detect VDJ mutations in plasma samples, Sarkozy et al. assessed the prognostic value of the ctDNA level at diagnosis from 34 patients with FL. In a multivariate analysis, authors found that a high baseline ctDNA level was the only independent factor significantly associated with a worse PFS (81). Similarly, Delfau-Larue et al. retrospectively analyzed baseline samples from 133 patients with FL using ddPCR and IgH rearrangements. A significant correlation was found between TMTV and both CTCs and cfDNA, with a worse PFS in patients with high cfDNA and TMTV (82).

MRD assessment by NGS can also help in predicting clinical outcomes in patients with FL. Recent studies suggested that ctDNA may be a more sensitive prognostic marker compared to radiographic response. Significantly shorter PFS was seen among patients with MRD positivity during, or after the end of treatment. In addition, MRD negativity at 1-year and 2-years post-treatment was associated with an improved PFS both in partial and complete remission (83, 84). A prospective study by Distler et al. also evaluated the ability of peripheral blood ctDNA by NGS to serially monitor untreated FL patients during watchful waiting. Baseline ctDNA significantly correlated with the TMTV on PET scan, and serial monitoring of ctDNA demonstrated patterns of sharp increases prior to progression. In non-progressing patients, there was an overall trend for decreasing ctDNA values over time, and undetectable ctDNA correlated with spontaneous clinical regression in some patients (85).

ctDNA may also serve as a minimally invasive marker for predicting FL progression and its transformation into diffuse large B-cell lymphoma. An analysis of tumor and peripheral blood samples from transformed FL patients identified *de novo* mutations that occurred at the time of transformation and often weeks to months prior (53). Ladetto at al showed that several MRD negative results over time reduced the likelihood of FL relapse, but more data into the extent of clinical relevance of serial monitoring is warranted (86).

MRD assessment in FL has been explored as a guide to therapeutic decision-making for additional therapy with Rituximab, which can convert MRD positive FL to MRD negative in cases such as advanced FL after chemotherapy and localized FL after radiotherapy. One of the main factors limiting the use of such monitoring in management is a lack of detectable molecular in a significant proportion of cases. Technical and technological advancement are underway to potentially overcome these limitations (87, 88).

### 3.5 Primary CNS lymphoma

Primary central nervous system lymphoma (PCNSL) is a rare but aggressive type of lymphoma with generally poor survival rates. The use of cfDNA and ctDNA as minimally invasive liquid biopsies has been studied in PCNSL as a way to improve the diagnosis and treatment of this anatomically challenging disease. A retrospective study on 25 patients with PCNSL demonstrated that somatic mutations and gene alterations can be detected by NGS-based circulating cfDNA sequencing (89). In patients with MYD88 L265P mutated PCNSL, several studies suggested the important role of detecting this mutation in plasma and cerebrospinal fluid (CSF) samples as an additional diagnostic tool (90–92). A study by Watanabe et al. showed that ddPCR-based detection of MYD88 mutation from CSF cfDNA was reliable and consistent with those of standard tumor-derived DNA (93). CSF ctDNA was also shown to be better than plasma ctDNA and flow cytometry at detecting CNS lesions and residual disease, hence its utility in predicting relapses (94).

Heger et al. recently found that the assessment of cfDNA fragment length profiles and their dynamic changes has the potential to significantly improve treatment guidance in PCNSL. Higher average mutated allele frequency in a plasma sample at baseline was associated with worse OS and PFS. Similarly, mutation-based tracking of peripheral residual disease showed that its persistence during treatment was significantly associated with reduced PFS and OS (95). Using NGS CAPP-seq, another study demonstrated the utility of ctDNA as a decision-making tool in PCNSL treatment guidance, by accurately mirroring tumor burden, serving as clinical biomarker for risk stratification, and predicting outcomes (96).

### 3.6 T-cell lymphoma

Adult T-cell leukemia/lymphoma (ATL) is a T-cell malignancy that arises in long-term carriers of human T-lymphotropic virus type 1. Being extremely aggressive, the survival of some subtypes is around 8 to 10 months. For the past 35 years, the average survival has remained unchanged (97). T-cell lymphoma is less common than B-cell lymphoma; hence, there is less evidence for the liquid biopsy in T-cell lymphoma. In addition, to diagnose the latter or to determine the mutational defect, allele-specific polymerase chain reaction (AS- PCR) can be used to identify HOAG17V/IDH2R172 mutations (17). However, no association was done between mutations and the clinical parameters nor survival in a study done by Hayashida et al. (98). In fact, in a study with 34 patients diagnosed with peripheral T-cell lymphoma where TCR rearrangements was detected by dPCR, Milkjovic et al. reported an average of 2.6-log decrease in the ctDNA levels and an early clearance of ctDNA after the first two cycles of treatment. However, the decrease was not associated with a statistically significant improvement in the event free survival (median (95% CI), 8.4 (0.1–NR) *vs*. 2.0 (0.1–NR) years; p = 0.32) or OS (median, 8.4 (0.3–NR) *vs*. 7.0 (0.5–NR) years; p = 0.44) (99). Therefore, due to the lack of association and evidence, the use of liquid biopsy for detection of minimal residual disease in T-cell lymphoma is still inconclusive.

### 3.7 Hodgkin lymphoma

Classical Hodgkin’s lymphoma (cHL) is a treatable malignancy with an annual incidence rate of 10% of new lymphomas and a prevalence rate of 1% of all cancers in the western part of the world. Multiagent chemotherapy (escalated BEACOPP, ABVD or brentuximab+AVD) and radiation therapies ensured an excellent outcome with a 5-year progression free survival rate of 65% to 95% (100, 101). Many patients, after only two cycles of frontline chemotherapy, benefit, so their treatment intensity gets de-escalated (102). However, around 25% of patient will relapse, shedding the lights on the urgency to understand the biological processes involved and to select useful biomarkers (101). To date, there is not yet pathognomonic biomarkers for classic Hodgkin lymphoma because Hodgkin Reed-Sternberg (HRS) tumor cells are very rare (0.1–3% of cells in the tissue) (103). Due to the low tumor cell count (around only 1% of lymphoma tissue), using tissue-based techniques to genotype HL is a challenging approach. Laser microdissection and flow sorting of the malignant HRS cells to molecularly profile HL has been used by previous studies, but these complex methods have resulted in less than 100 HL genomes being profiled to date (5). Recent research has proved that ctDNA analysis leads to more comprehensive genomic characterization of cHL (104). Two studies assessing the use of liquid biopsies to genotype HL were published (105). The studies focused on the feasibility of liquid biopsy in genotyping HL and identified the most recurrent mutated genes, such as STAT6, GNA13, ITPKB, SOCS1 and TNFAIP3 (106). In fact, using CAPP-seq on cfDNA, Spina et al. proved that STAT6, TNFAIP3, ITPKB, GNA13, B2M, ATM, SPEN, and XPO1 are the most mutated genes (105). Concerning the MRD, Camus et al. explained that the XPO1 E571K mutation will aid as a biomarker in cHL, by means of digital PCR. Their study presented a not statistically significant association claiming that patients with a detectable XPO1 mutation at the end of treatment could have a shorter progression free survival (107). To conclude, the limitations of these studies are numerous, and more data is crucial prior to integrating liquid biopsies in monitoring MRD in lymphomas (99).

### 3.8 Chronic lymphocytic leukemia

Chronic lymphocytic leukemia (CLL) is the most common lymphoproliferative disease in the elderly of the western world, and it has a multitude of clinical outcomes. In CLL, even though the MRD is not yet well investigated as in other hematologic tumors, recent studies are revealing its role in providing better progression-free and overall survival rates (92). Relying on ctDNA for disease observing has also recently been established in CLL (108). Multiparameter flow cytometer (MFC) and real time quantitative polymerase chain reaction (RQ-PCR) are the most common used techniques. Even though CLL is a circulation disease in the peripheral blood, much research has proven the effectiveness of ctDNA, using digital PCR and targeted sequencing. In CLL, ctDNA analysis display fluctuations in the disease across tissues, including the bone marrow and lymph nodes. This offers supplementary information that cannot be attained by only monitoring the circulating disease. Moreover, in CLL, serial ctDNA investigation favors examining clonal dynamics and discovering genomic changes linked with Richter’s syndrome (104). To conclude, additional data and research ought to be performed to implement the MRD monitoring as a harmonizing method additionally to the ones used.

### 3.9 Multiple myeloma

The standard method for determining a patient’s diagnosis, prognosis, and genetic assessment in MM is a BM aspirate and biopsy and performing fluorescent *in situ* hybridization (FISH). However, because it necessitates an invasive approach, this uncomfortable method provides a significant disadvantage for routine illness assessment. New research also showed that a single BM aspirate is unable to display the complex genetic heterogeneity of MM (109). Liquid biopsies may represent an emerging tool that can be used in clinical settings to detect and track MM, leading to a more specific treatment approach (110).

Currently, techniques utilized to detect MRD comprise of next-generation flow cytometry (NGF) and NGS. The initial technique used to assess MRD was flow cytometry more than a decade ago, which had a sensitivity of 10^−4^ initially and has now improved to 2×10^−6^ (111). The EuroFlow consortium has standardized this methodology (112). Alongside, NGS has also emerged for measuring MRD, where immunoglobulin gene segments are amplified and sequenced with consensus primers (113). The Adaptive Clonoseq platform (formerly known as LymphoSIGHT) presently has a sensitivity of 6.77×10^−7^, using 20 μg DNA from 1mL of bone marrow aspirate (114, 115).

There is a high level of agreement between NGF and NGS techniques in detecting MRD, exceeding 80% in newly diagnosed patients as shown in the FORTE (116) and CASSIOPEIA (117) trials. The concordance was similarly high, ranging from 85.8% to 92.9% when comparing NGF with NGS from a different platform called LymphoTrack. The choice of MRD assay typically depends on institutional preference and availability. However, NGS requires a baseline sample to provide a trackable sequence, which is not necessary for NGF. In some cases, a trackable sequence for NGS could not be identified in 7.8% of samples (118). Another advantage of NGF is that it can detect hemodilution by examining mast cell, erythroblast, and B-cell precursor populations (112). Moreover, NGF can potentially assess the bone marrow microenvironment, which may have prognostic relevance in research settings.

Several meta-analyses have demonstrated that achieving a deeper response beyond complete remission (CR) is linked to an improvement in OS (119–121). The initial meta-analyses focused on transplant-eligible patients who underwent intensive therapy and where MRD was mostly evaluated by older and less sensitive flow cytometry (10^−4^). However, a recent meta-analysis builds upon these prior observations and includes older, transplant-ineligible patients, as well as patients with relapsed disease. The study revealed that compared to MRD-positive disease, MRD-negative status was associated with better progression-free survival (hazard ratio 0.33; 95% CI 0.29-0.37) and OS (hazard ratio 0.45; 95% CI 0.39-0.51) across various patient populations, including those with relapsed or high-risk disease (121). It is noteworthy that MRD status can also stratify patients in CR, with an OS of 112 months for MRD-negative patients, compared to 82 months for MRD-positive patients (120).

Considering these discoveries, the status of MRD is being used more often as a benchmark to compare different treatment plans, particularly as these plans are becoming more successful in achieving deeper responses. The International Myeloma Working Group and Bone Marrow Transplant Clinical Trials Network have established definitions for MRD and its measurement, with the former recommending a sensitivity of 10^-5^ (40, 122). The use of MRD as a replacement endpoint for regulatory purposes is currently being debated (123) and is being explored by a coalition of academic organizations and pharmaceutical companies known as the International Independent Team for Endpoint Approval of Myeloma MRD (124–126).

Diving into how MRD can be utilized, one should notice that nearly immediately following the onset of the premalignant clonal activity, CTCs can be detected. These cells are liberated from the BM into the circulation and typically join other BM sites (127). In fact, Sata et al. found that polymerase chain reaction using allele-specific oligonucleotides (ASO-PCR) levels in BM cells and peripheral blood cells were strongly correlated, which suggests that MM cells may circulate in the peripheral blood (128). CTCs can be found in monoclonal gammopathy of undetermined significance (MGUS), smoldering multiple myeloma (SMM) and overt MM. The primary difference between these disorders is the CTC rate, which rises in percentages from 0.0002% for MGUS to 0.004% for SMM and 0.04% for overt MM (129). Interestingly, a high CTC count in SMM was associated with a significant likelihood of progressing to clinical MM within 2–3 years after diagnosis (130). Notably, the CTC count was thought to be a distinct prognostic factor. Lower progression-free survival (PFS) related to rising numbers of CTCs. In addition to that, MM patients with undetectable CTCs exhibited exceptionally long PFS (131). By investigating the clonotypic VDJ rearrangement for tracking circulating MM cells and cell-free myeloma DNA, Oberle et al. found that most non-responders and progressors had lingering myeloma cells and cell-free myeloma DNA in the blood (132).

The extension and aggressiveness of the illness appear to be related to cfDNA levels. MM patients without extramedullary involvement and extramedullary MM (EMM) patients’ plasma samples were used in a study to assess the relationship between cfDNA and MM progression. Surprisingly, individuals with extramedullary involvement produced higher levels of cfDNA than participants without this sort of involvement. Additionally, compared to BM aspirates, plasma tumor-originated cfDNA (ctDNA) showed a higher concordance to extramedullary tumors. Considering this, cfDNA is a helpful alternative for EMM genetic identification, particularly when the tumor is difficult to access. It may also be used to monitor MM progression (133). Numerous studies have looked at the genetic traits in SMM that can reliably identify patients with high risk SMM. According to certain findings, cfDNA levels were lower in SMM participants than in patients with overt MM. A study looked at whether variations in the quantity of cfDNA correlate with risk levels identified by the 70 gene expression profile (GEP70). The amounts of cfDNA were noticeably higher in the GEP70 high-risk group compared to the low risk set (134). Another study demonstrated that cfDNA may be used to determine the tumor burden and forecast the likelihood of a disease recurrence in MM patients. Additionally, cfDNA with driver mutations provided more reliable evidence of the recurrence of MM than serum free light chains (135). According to Rustad et al., there is a correlation between the quantity of mutated alleles in the peripheral blood and the percentage of BM plasma cells, which may reflect mutated cells, tumor load, and the development of a more advanced disease (136).

There are few papers that compare the assessment of the minimal residual disease (MRD) using cfDNA to other common approaches including flow cytometry and DNA assessment in the BM (137). Mazzotti et al. demonstrated, using solely IgH gene rearrangements, that there is no relationship between DNA and BM for the MRD by NGS (137). Another research showed that cfDNA may be used to assess the MRD, particularly in EMM. Although a pertinent link between the quantity of tumor-specific cfDNA and clinical outcomes has been found in other studies, the findings in the case of the MRD assessment were not significant (138). As of now, using cfDNA alone has little value in assessing the MRD in MM subjects, despite mounting evidence that it may be a helpful auxiliary method for the treatment and control of the disease and, possibly, with further development, becoming a useful tool for assessing the MRD (139). The clonoSEQ^®^ Assay (Adaptive Biotechnologies Corporation, Seattle, USA) identifies and tracks unique disease-associated immunoglobulin (Ig) sequences by next-generation sequencing of IgH, IgK, and IgL rearrangements and IgH-BCL1/2 translocations in malignant B cells and is useful for MRD assessment in CLL, ALL and Multiple Myeloma (MM). The MRD assessment using clonoSEQ^®^ can be used in the assessment of myeloma burden throughout treatment with accuracy, sensitivity and standardization when compared to traditional cytomorphology. Both bone marrow based and peripheral blood based assays are available (115).

Different studies explored liquid biopsies in MM. *KRAS, NRAS* and *BRAF* mutations were evaluated by both Mithraprabhu et al. and Kis et al., in 2017, The former found that the analysis of ctDNA in seven patients indicated that the frequency of certain mutant clones increased at the same time as clinical relapse, suggesting that it could be used as a non-invasive method to monitor the progression of MM disease (140). The latter found that the concentration of cfDNA was significantly higher in the MM cohort than in 56 patients with advanced solid tumors (p < 0.001). The concentration of cfDNA was associated with advanced disease, as shown by a correlation with late relapse compared to early relapse (p = 0.016). The frequency of mutations in cfDNA showed strong agreement with those in bone marrow samples, with R^2^ values ranging from 0.913 to 0.997 (141). Gerber et al. evaluated somatic mutations utilizing CAPP-seq. The quantity of cfDNA was associated with clinical and pathological indicators that reflect the size and spread of the tumor, such as the degree of bone marrow plasma cell infiltration (p = 0.02). The frequencies of variant alleles in plasma samples were positively correlated with those in tumor biopsies (rs = 0.58, p = 9.6 × 10^−5^) (142). **Table 2** represents trials evaluating MRD to guide treatment.

**Table 2:** Trials utilizing MRD to guide initial and maintenance therapy.

Study	Primary endpoint	Population	Treatment administered	Based on
1. MRD testing utilizing NGF (10^-5^)				
RADAR	PFS	Standard risk patients after auto SCT	Randomization to arms including R maint, RVd consolidation + R maint, R-isa maint, or isa-RVd consolidation + R isa maint	Patients who received Isa main were MRD -ve after initial treatment. The remaining tested +ve.
REMNANT	PFS	MRD -ve patients randomized to two arms	Dara-Kd	Patients who received Dara-Kd tested MRD +ve *vs* those who were only continuously observed every 4 months
NCT04221178	MRD -ve at one year	Patients on 3 years of continuous maint	Maint treatment	None of the patients tested MRD +ve
PREDATOR	EFS	Patients after 1-2 prior lines of therapy who are MRD negative	Dara *vs*. observation	Patients who were on Dara tested MRD+ve while those on observation were MRD-ve
2. MRD testing utilizing NGS				
NCT04140162	MRD	Patients who got induction with dara-R	Following induction, received either consolidation with dara-RVd *vs*. maint dara	Patients on dara-RVd tested +ve *vs*.-ve for those who received maint dara
AURGIA*	MRD	Patients who got auto-SCT	Patients received DARA-R *vs* R only.	Patients on DARA-R only tested MRD+ve.
MASTER*	MRD	Patients who received dara-KRd	Additional dara_KRd *vs*. observation after achieved MRD-ve twice	Patients receiving additional dara-KRd were those who tested MRD+ve.

EFS, event-free survival.

*denotes (10^-5^).

### 3.10 Waldenström’s macroglobulinemia

Waldenström’s macroglobulinemia (WM) is a type of lymphoplasmacytic lymphoma with recurring somatic mutations, including MYD88 and CXCR4 mutations, which can be found in up to 97% and 40% of cases, respectively (143). While the current genomic analysis of CD19+ selected cells from bone marrow aspirates is the standard procedure, liquid biopsies are becoming a reliable alternative. Bagratuni et al. collected and analyzed approximately 100 paired bone marrow and peripheral blood samples from patients with IgM monoclonal gammopathies (144). They found that cfDNA is a reliable biomarker for the detection of MYD88 and CXCR4 mutations, with high concordance rates between bone marrow and cfDNA detection across all stages of the disease (145). Drandi et al. also demonstrated the feasibility of detecting MYD88 mutations in cfDNA using droplet digital PCR (ddPCR), with good concordance rates between bone marrow and cfDNA detection. This is of notable clinical importance since the MYD88^L265P^ is a diagnostic and predictive biomarker of therapy response. Diagnostically, liquid biopsies including surface marker and genetic mutations such as MYD88^L265P^ participate in distinguishing WM from B-cell lymphoproliferative disorders as well as from related IgM monoclonal gammopathies. Furthermore, MYD88^L265P^ mutation detection have a role in MRD monitoring as well. Dynamics of MRD clearance need to be explored further in a controlled prospective setting to draw conclusions in comparing optimal sources of sampling, but initial retrospective results suggest the mutation clears from the PB earlier than BM (146). The ddPCR technique can also be applied in other liquid biopsies, such as the detection of the MYD88^L265P^ mutation in the cerebrospinal fluid of patients with suspected Bing-Neel syndrome (147). Overall, liquid biopsies have the potential to serve as a reliable alternative to bone marrow CD19+ selected cell analysis.

### 3.11 Chronic myelocytic leukemia

Different groups have recently conducted tests on the quantification of BCR-ABL1 transcripts by using digital PCR in patients with chronic myeloid leukemia (CML). One such study, known as the ISAV study, involved CML patients who had been on first-generation TKI therapy for more than two years and had undetectable BCR-ABL1 levels by RT-qPCR for at least 18 months. The researchers, including Mori et al., used the Fluidigm dPCR platform, which involves multiple parallel micro-reactions, to select patients who were suitable for discontinuing TKIs. The study showed that most patients who remained in a stable state of undetectable minimal residual disease (MRD) by RT-qPCR were dPCR-negative at the time of TKI suspension (148). Furthermore, the majority of patients who relapsed and were unable to sustain TKI discontinuation were found to be dPCR-positive (68% *vs*. 43%) (149, 150). Another meta-analysis study also demonstrated the effectiveness of dPCR in supporting the selection of CML patients for TKI discontinuation. Other groups have also confirmed the accuracy and sensitivity of first-generation dPCR platforms in measuring BCR-ABL1 transcripts (151).

Goh and their colleagues assessed the ability of nanofluidic dPCR to monitor the continual decrease in BCR-ABL1 transcript levels, even after it had become undetectable using traditional RT-qPCR methods. They found that dPCR had a 2-3 log improvement in sensitivity over RT-qPCR, with 75% of samples that were undetectable by RT-qPCR showing positivity for BCR-ABL1 transcripts when screened by dPCR (152). Other studies have also confirmed the improved sensitivity of dPCR. In a comparison between RT-qPCR and dPCR, the two methods had a 99% correlation, but only dPCR was able to predict a logarithmic increase in MRD. Additionally, dPCR was able to detect an increase in BCR-ABL1 transcripts up to three months earlier than RT-qPCR (153). The use of dPCR for BCR-ABL1 quantification has also been explored in pediatric CML cases. In these cases, dPCR improved MRD monitoring by detecting the BCR-ABL1 fusion gene on genomic DNA. This is because in pediatric patients, the ABL1 and BCR breakpoint cluster regions are positioned in a way that affects the match between the primers and probe on the BCR-ABL1 transcript (154). This evidence was confirmed by another study, which demonstrated that monitoring MRD through the detection of both cDNA and gDNA is the most sensitive approach for pediatric CML patients (155).

It is possible to detect BCR-ABL1 on gDNA instead of cDNA in adults, even if they have a long history of undetectable MRD using conventional RT-qPCR based on RNA analysis (155). This is because the fusion gene may not be transcribed. A gDNA-based dPCR approach can accurately measure the major breakpoint region and the presence of the BCR-ABL1 fusion gene, increasing sensitivity compared to fluorescence *in situ* hybridization (156, 157). A second-generation chip-based dPCR platform has been developed in recent years for the detection of BCR-ABL1 transcripts. Bernardi and their colleagues demonstrated that dPCR can provide precise, sensitive, and accurate quantification of BCR-ABL1 transcripts in different biological matrices, including the gold standard peripheral blood cells. They also showed that CML cells can release EVs (158, 159). The presence of BCR-ABL1 positive extracellular vesicles (EVs) has been shown to have an impact on both *in vitro* and *in vivo* tumor progression. However, the biological significance of these EVs has not yet been fully understood. CML patients have been found to have a higher number of circulating EVs at diagnosis compared to patients in early stages of treatment or in “deep” MRD, as well as healthy individuals. Additionally, the presence of BCR-ABL1 transcripts detected by RT-qPCR has only been observed in the cargo of exosomes isolated from CML patients in the early stages of the disease. With the application of dPCR, it has been possible to detect the BCR-ABL1 transcript in patients with undetectable MRD levels, using a new biological substrate (circulating exosomes) and avoiding invasive clinical procedures. This suggests the potential of using dPCR to detect active leukemic cells in a less invasive way (158).

Using second-generation dPCR platforms has allowed for the possibility of suspending TKIs therapy for CML patients (160, 161). Italian and French cooperative groups have investigated the use of dPCR in this context and have found that BCR-ABL1 values measured by dPCR are a significant predictor of molecular recurrence. This finding has been supported by a meta-analysis of five different trials (162–164). Additionally, dPCR is reported to be more reliable than RT-qPCR in amplifying all transcript variants present in CML patients. Third-generation dPCR chip-based platforms, such as novel microfluidic array partitioning consumable devices, have also been used to precisely quantify BCR-ABL1 transcripts down to a 0.01% allele frequency with high reproducibility across many replicates, providing further evidence of dPCR’s ability to overcome RT-qPCR limitations (165).

As a result of the promising outcomes, it is not surprising that new commercial assays certified for diagnostic use have emerged for detecting BCR-ABL1 transcripts by dPCR. Furthermore, some expert panels have recommended coordinated international efforts to standardize MRD monitoring by dPCR in adult CML patients through inter- and intra-laboratory tests (166). This push will support the routine use of dPCR alongside RT-qPCR in some settings of CML patients. The routine use of dPCR is preferred over other technologies due to its lower analysis costs, ranging from EUR 4 to 12 per sample, making it a more affordable option (166).

The CD26 antigen is preferentially expressed on leukemic cells from CML patients but not on normal BM cells or those from other neoplasms (167). In one study, CD26+ leukemic stem cells (LSC) were present in around 30% of CML patients with undetectable BCR-ABL1 by RQ-PCR. Quiescent residual LSC may not express BCR-ABL1 transcriptionally, which makes them undetectable by RQ-PCR or by other molecular techniques investigating the BCR-ABL1 transcript. This opens the opportunity to use residual CD26+ LSC in the PB by flow cytometry for MRD monitoring, in addition to standard BCR-ABL1 detection. This is being investigated further (168).

Other methods of BCR-ABL1 independent MRD monitoring currently under investigation for potential clinical use include miRNA quantification and mitochondrial DNA (mtDNA) mutations detection (169).

## 4 Artificial intelligence and liquid biopsies

Artificial Intelligence (AI) will inescapably be involved in the future landscape of hematological disease management at all stages. Different models using AI have already shown promise in several areas of hematology. For instance, in cytomorphology AI models were able to differentiate cell types and detect malignant clones. In cytogenetics, AI models were able to support karyotyping (170). Technologies leveraging AI have been rapidly evolving over the last few years and may very well be on their way to enhance the clinical practice of the human hematologist. In fact, in 2022 alone, the FDA has approved 91 new additions to their list of legally marketed AI/ML-enabled medical devices (171).

Although the terms “AI” and “machine learning” (ML) are commonly used interchangeably in medical literature, ML is actually a subset of AI that refers to the automatic detection of patterns and associations within the data analyzed. AI can be broadly separated into two groups: strong and narrow. Narrow or weak AI performs one task based on a defined dataset, such as the automatic classification of cell types, and refers to all current medical ML-based algorithms. To qualify as strong, the AI model would have to reliably perform multiple tasks without the need for human intervention, but none of the present-day models achieve this in medicine, ethical and legal questions aside (172).

Phenotypic assessment of peripheral blood (PB) and bone marrow (BM) samples is still the backbone of hematological diagnostics and depends on the experience and capabilities of hematologists and pathologists with limited reproducibility. Several models have been created to classify blood cell types and morphological features, even reaching sensitivity and specificity of over 93.5% and 96.0% respectively (173). For instance, one model was able to reliably identify acute promyelocytic leukemia, an oncologic emergency, in AML samples in a fraction of the time it would take using traditional methods (174). There is even an ongoing prospective clinical trial (ClinicalTrials.gov Identifier: NCT04466059) leveraging an AI model to classify single-cell images of PB smears side-by-side to routine diagnostics with sensitivity and specificity of AI-guided diagnostics as the primary outcomes measured. In TKI treatment selection of CML chronic phase patients, the choice between several highly effective treatment options is complex and frequently subjective. An AI model developed by Sasaki et al. was able to give personalized recommendations that were associated with better survival probability compared to treatment with a non-recommended therapy (175).

MRD has been proving to be of increasing utility in multiple hematological diseases. Multiparameter flow cytometry (MFC) is routinely used to measure MRD in the clinical setting, but it strongly depends on operator skill and expert knowledge. In AML and MDS patients, Ko et al. were able to apply supervised ML techniques to create an automated MFC interpretation algorithm that objectively, reliably, and rapidly detected MRD with high accuracy (> 90%), thus opening the door to the resolution of inter-lab standardization issues (176). Similarly, in childhood B-ALL, Reiter et al. showed high correlation between operator-based and their automated MFC MRD assessment (177). In CLL, Salama et al. created a working model that detected MRD with high accuracy, albeit needing additional clinical validation (178).

Moreover, Hoffman et al. developed an AI algorithm that aims to prevent technique-related false-negative AML MRD results due to critical BM dilution with PB (179). Going even further, Guerrero et al. developed a model to predict MRD negativity and therefore survival outcomes in patients with newly diagnosed multiple myeloma, based on tumor and immune biomarkers (180).

Concerning future considerations although AI-based methods have shown promise and success, both the medical community and the public have not been persuaded enough to embrace AI in routine clinical practice. This of trust in AI predictions can begin to be tackled by focusing on explainable and widely validated models (181). Currently, AI still mostly exists in the research setting, but the future of hematology research will be the integration of the swaths of data from research and the real-world data to find the best way to allow AI to expand the work of human physicians to a point none could reach individually (182).

## 5 Conclusion

Liquid biopsy for MRD testing using blood is rapidly evolving in the field of lymphoid malignancies. Most advancement is seen in the evaluation, management and in the post-therapy monitoring of ALL leading to routine clinical utilization. Liquid biopsy based MRD testing in common non-Hodgkin’s lymphoma such as diffuse large B cell lymphoma, follicular or mantel cell lymphoma remains experimental. Similarly, NGS based MRD testing in Hodgkin’s lymphoma is used in the research setting only for now. In the field of CLL and multiple myeloma NGS based MRD testing has been widely utilized in clinical trials and has started to come to the clinic for patient evaluation after therapy. MRD guided approach to therapy will probably be the standard of care moving forward in hematologic malignancies. Finally, employing artificial intelligence may simplify the complexities associated with current high-tech, high-skill and labor-intensive process of MRD testing using liquid biopsy. AI based algorithm can have the potential to prevent technique-related false-negatives or false positives.

## Author contributions

MaZ drafted the manuscript. JK, LS, EH, MoZ, SA, WM, MM, SM and MaZ contributed to the discussion section. CC conceived the idea for the paper. All authors contributed to the article and approved the submitted version.

## Glossary

**Table:**

AI	Artificial Intelligence
ALL	Acute Lymphoblastic Leukemia
AML	Acute Myeloid Leukemia
ATL	Adult T-cell leukemia/lymphoma
AS-PCR	Allele-specific polymerase chain reaction
BM	Bone Marrow
cfDNA	Cell-free DNA
cHL	Classical Hodgkin&rsquo;s lymphoma
CLL	Chronic lymphocytic leukemia
CML	Chronic Myeloid Leukemia
CSF	Cerebrospinal fluid
CTC	Circulating tumor cells
ctDNA	circulating tumor DNA
DLBCL	Diffuse Large B cell Lymphoma
ddPCR	Droplet Digital PCR
FL	Follicular cell lymphoma
HRS	Hodgkin Reed-Sternberg
IG	Immunoglobulin
MCL	Mantle cell lymphoma
MDSs	myelodysplastic syndromes
MFC	Multiparameter flow cytometer
miRNAs	MicroRNAs
ML	Machine Learning
MRD	Measurable Residual Disease
mtDNA	Mitochondrial DNA
MPNs	myeloproliferative neoplasms
NGS	Next-Generation Sequencing
OS	Overall survival
PB	Peripheral Blood
PFS	Progression-free survival
PCR	Polymerase chain reaction
PCNSL	Primary central nervous system lymphoma
PhasED-Seq	Phased variant enrichment and detection sequencing
qRT-dPCR	real-time quantitative digital polymerase chain reaction
RT-PCR	Reverse transcriptase PCR
RQ-PCR	Real time quantitative polymerase chain reaction
SCT	Stem-cell transplant
SVs	Structural Variants
TCR	T-cell receptor
TDEs	Tumor-derived exosomes
TMTV	Total metabolic tumor volume
MRD	Minimal Residual Disease

## References

[1] Chen M Zhao H. Next-generation sequencing in liquid biopsy: cancer screening and early detection. Hum Genomics (2019) 13(1):1–10. doi: 10.1186/s40246-019-0220-8 31370908 PMC6669976

[2] Deng Z Wu S Wang Y Shi D. Circulating tumor cell isolation for cancer diagnosis and prognosis. EBioMedicine (2022) 83:104237. doi: 10.1016/j.ebiom.2022.104237 36041264 PMC9440384

[3] Lin D Shen L Luo M Zhang K Li J Yang Q. Circulating tumor cells: biology and clinical significance. Signal Transduction Targeted Ther (2021) 6(1):1–24. doi: 10.1038/s41392-021-00817-8 PMC8606574 34803167

[4] Gharbaran R Park J Kim C Goy A Suh KS. Circulating tumor cells in hodgkin’s lymphoma–a review of the spread of HL tumor cells or their putative precursors by lymphatic and hematogenous means, and their prognostic significance. Crit Rev Oncol Hematol (2014) 89(3):404–17. doi: 10.1016/j.critrevonc.2013.09.004 24176672

[5] Cirillo M Craig AF Borchmann S Kurtz DM. Liquid biopsy in lymphoma: molecular methods and clinical applications. Cancer Treat Rev (2020) 91:102106. doi: 10.1016/j.ctrv.2020.102106 33049623 PMC8043056

[6] Gai W Sun K. Epigenetic biomarkers in cell-free DNA and applications in liquid biopsy. Genes (2019) 10(1):32. doi: 10.3390/genes10010032 30634483 PMC6356936

[7] Leon S Shapiro B Sklaroff D Yaros M. Free DNA in the serum of cancer patients and the effect of therapy. Cancer Res (1977) 37(3):646–50. 837366

[8] van der Vaart M Pretorius PJ. The origin of circulating free DNA. Clin Chem (2007) 53(12):2215–. doi: 10.1373/clinchem.2007.092734 18267930

[9] Stroun M Lyautey J Lederrey C Olson-Sand A Anker P. About the possible origin and mechanism of circulating DNA: apoptosis and active DNA release. Clin Chim Acta (2001) 313(1-2):139–42. doi: 10.1016/S0009-8981(01)00665-9 11694251

[10] Khier S Lohan L. Kinetics of circulating cell-free DNA for biomedical applications: critical appraisal of the literature. Future Sci OA (2018) 4(4):FSO295. doi: 10.4155/fsoa-2017-0140 29682327 PMC5905581

[11] DeWeerdt S. The origins of ovarian cancer. Nature (2021) 600(7889):S42–S4. doi: 10.1038/d41586-021-03717-7

[12] Sun K Jiang P Chan KA Wong J Cheng YK Liang RH. Plasma DNA tissue mapping by genome-wide methylation sequencing for noninvasive prenatal, cancer, and transplantation assessments. Proc Natl Acad Sci (2015) 112(40):E5503–E12. doi: 10.1073/pnas.1508736112 PMC4603482 26392541

[13] Bobkov YV Walker WB III Cattaneo AM. Altered functional properties of the codling moth orco mutagenized in the intracellular loop-3. Sci Rep (2021) 11(1):1–16. doi: 10.1038/s41598-021-83024-3 33594162 PMC7887336

[14] Heitzer E Auer M Ulz P Geigl JB Speicher MR. Circulating tumor cells and DNA as liquid biopsies. Genome Med (2013) 5(8):1–11. doi: 10.1186/gm477 23998943 PMC3979149

[15] Frattini M Gallino G Signoroni S Balestra D Lusa L Battaglia L. Quantitative and qualitative characterization of plasma DNA identifies primary and recurrent colorectal cancer. Cancer Lett (2008) 263(2):170–81. doi: 10.1016/j.canlet.2008.03.021 18395974

[16] Barbeau D Persoons R Marques M Hervé C Laffitte-Rigaud G Maitre A. Relevance of urinary 3-hydroxybenzo (a) pyrene and 1-hydroxypyrene to assess exposure to carcinogenic polycyclic aromatic hydrocarbon mixtures in metallurgy workers. Ann Occup Hyg (2014) 58(5):579–90. doi: 10.1093/annhyg/meu004 PMC4305110 24504174

[17] Colmenares R Álvarez N Barrio S Martínez-López J Ayala R. The minimal residual disease using liquid biopsies in hematological malignancies. Cancers (Basel) (2022) 14(5):1310. doi: 10.3390/cancers14051310 35267616 PMC8909350

[18] Wurdinger T. Tumor-educated platelets. Blood (2019) 133(22):2359–64. doi: 10.1182/blood-2018-12-852830 30833413

[19] Roweth HG Battinelli EM. Lessons to learn from tumor-educated platelets. Blood (2021) 137(23):3174–80. doi: 10.1182/blood.2019003976 PMC8351883 33940594

[20] Biting Z Kailun X Zheng X Chen T Wang J Yongmao S. Application of exosomes as liquid biopsy in clinical diagnosis. Signal Transduction Targeted Ther (2020) 5(1). doi: 10.1038/s41392-020-00258-9 PMC7400738 32747657

[21] Yu D Li Y Wang M Gu J Xu W Cai H. Exosomes as a new frontier of cancer liquid biopsy. Mol Cancer (2022) 21(1):1–33. doi: 10.1186/s12943-022-01509-9 35180868 PMC8855550

[22] Giannopoulou L Zavridou M Kasimir-Bauer S Lianidou ES. Liquid biopsy in ovarian cancer: the potential of circulating miRNAs and exosomes. Trans Res (2019) 205:77–91. doi: 10.1016/j.trsl.2018.10.003 30391474

[23] Budakoti M Panwar AS Molpa D Singh RK Büsselberg D Mishra AP. Micro-RNA: the darkhorse of cancer. Cell Signal (2021) 83:109995. doi: 10.1016/j.cellsig.2021.109995 33785398

[24] Montani F Bianchi F. Circulating cancer biomarkers: the macro-revolution of the micro-RNA. EBioMedicine (2016) 5:4–6. doi: 10.1016/j.ebiom.2016.02.038 27077096 PMC4816844

[25] Wang J Ni J Beretov J Thompson J Graham P Li Y. Exosomal microRNAs as liquid biopsy biomarkers in prostate cancer. Crit Rev Oncol Hematol (2020) 145:102860. doi: 10.1016/j.critrevonc.2019.102860 31874447

[26] Salehi M Sharifi M. Exosomal miRNAs as novel cancer biomarkers: challenges and opportunities. J Cell Physiol (2018) 233(9):6370–80. doi: 10.1002/jcp.26481 PMC13482446 29323722

[27] van Dongen JJ van der Velden VH Brüggemann M Orfao A. Minimal residual disease diagnostics in acute lymphoblastic leukemia: need for sensitive, fast, and standardized technologies. Blood J Am Soc Hematol (2015) 125(26):3996–4009. doi: 10.1182/blood-2015-03-580027 PMC4490298 25999452

[28] Sánchez R Ayala R Martínez-López J. Minimal residual disease monitoring with next-generation sequencing methodologies in hematological malignancies. Int J Mol Sci (2019) 20(11):2832. doi: 10.3390/ijms20112832 31185671 PMC6600313

[29] Brüggemann M Kotrova M. Minimal residual disease in adult ALL: technical aspects and implications for correct clinical interpretation. In: Hematology 2014, the American society of hematology education program book (2021 L Street, NW, Suite 900 Washington, DC 20036, USA: American Society of Hematology), vol. 2017. (2017). p. 13–21. 10.1182/asheducation-2017.1.13 PMC6142572 29222232

[30] Campana D. Determination of minimal residual disease in leukaemia patients. Br J Haematol (2003) 121(6):823–38. doi: 10.1046/j.1365-2141.2003.04393.x 12786792

[31] Faderl S Estrov Z. Residual disease in acute lymphoblastic leukemia of childhood: methods of detection and clinical relevance. Cytokines Cell Mol Ther (1998) 4(2):73–85. 9681246

[32] Van Dongen J Breit T Adriaansen H Beishuizen A Hooijkaas H. Detection of minimal residual disease in acute leukemia by immunological marker analysis and polymerase chain reaction. Leukemia (1992) 6:47–59. 1548936

[33] Honoré N Galot R van Marcke C Limaye N Machiels J-P. Liquid biopsy to detect minimal residual disease: methodology and impact. Cancers (Basel) (2021) 13(21):5364. doi: 10.3390/cancers13215364 34771526 PMC8582541

[34] Shin S Woo HI Kim J-W Kim Y Lee K-A. Clinical practice guidelines for pre-analytical procedures of plasma epidermal growth factor receptor variant testing. Ann Lab Med (2022) 42(2):141–9. doi: 10.3343/alm.2022.42.2.141 PMC8548242 34635607

[35] Pantel K Alix-Panabières C. Liquid biopsy and minimal residual disease–latest advances and implications for cure. Nat Rev Clin Oncol (2019) 16(7):409–24. doi: 10.1038/s41571-019-0187-3 30796368

[36] Della Starza I Chiaretti S De Propris MS Elia L Cavalli M De Novi LA. Minimal residual disease in acute lymphoblastic leukemia: technical and clinical advances. Front Oncol (2019) 9:726. doi: 10.3389/fonc.2019.00726 31448230 PMC6692455

[37] Galimberti S Genuardi E Mazziotta F Iovino L Morabito F Grassi S. The minimal residual disease in non-hodgkin’s lymphomas: from the laboratory to the clinical practice. Front Oncol (2019) 9:528. doi: 10.3389/fonc.2019.00528 31293969 PMC6606710

[38] Hoppe RT Advani RH Ai WZ Ambinder RF Aoun P Bello CM. Hodgkin Lymphoma, version 2.2012 featured updates to the NCCN guidelines. J Natl Compr Canc Netw (2012) 10(5):589–97. doi: 10.6004/jnccn.2012.0061 22570290

[39] Fürstenau M De Silva N Eichhorst B Hallek M. Minimal residual disease assessment in CLL: ready for use in clinical routine? Hemasphere (2019) 3(5). doi: 10.1097/HS9.0000000000000287 PMC6919470 31942542

[40] Kumar S Paiva B Anderson KC Durie B Landgren O Moreau P. International myeloma working group consensus criteria for response and minimal residual disease assessment in multiple myeloma. Lancet Oncol (2016) 17(8):e328–e46. doi: 10.1016/S1470-2045(16)30206-6 27511158

[41] Toft N Birgens H Abrahamsson J Griskevicius L Hallbook H Heyman M. Results of NOPHO ALL2008 treatment for patients aged 1-45 years with acute lymphoblastic leukemia. Leukemia (2018) 32(3):606–15. doi: 10.1038/leu.2017.265 28819280

[42] Kruse A Abdel-Azim N Kim HN Ruan Y Phan V Ogana H. Minimal residual disease detection in acute lymphoblastic leukemia. Int J Mol Sci (2020) 21(3). doi: 10.3390/ijms21031054 PMC7037356 32033444

[43] Short NJ Jabbour E Albitar M de Lima M Gore L Jorgensen J. Recommendations for the assessment and management of measurable residual disease in adults with acute lymphoblastic leukemia: a consensus of north American experts. Am J Hematol (2019) 94(2):257–65. doi: 10.1002/ajh.25338 PMC6572728 30394566

[44] Wood B Wu D Crossley B Dai Y Williamson D Gawad C. Measurable residual disease detection by high-throughput sequencing improves risk stratification for pediatric b-ALL. Blood (2018) 131(12):1350–9. doi: 10.1182/blood-2017-09-806521 PMC5865233 29284596

[45] Key Statistics for Acute Lymphocytic Leukemia (ALL). American Cancer society (2022) (Accessed January 12, 2022).

[46] Schwarz AK Stanulla M Cario G Flohr T Sutton R Moricke A. Quantification of free total plasma DNA and minimal residual disease detection in the plasma of children with acute lymphoblastic leukemia. Ann Hematol (2009) 88(9):897–905. doi: 10.1007/s00277-009-0698-6 19165483

[47] Arthur C Rezayee F Mogensen N Saft L Rosenquist R Nordenskjold M. Patient-specific assays based on whole-genome sequencing data to measure residual disease in children with acute lymphoblastic leukemia: a proof of concept study. Front Oncol (2022) 12:899325. doi: 10.3389/fonc.2022.899325 35865473 PMC9296121

[48] van der Velden VH Jacobs DC Wijkhuijs AJ Comans-Bitter WM Willemse MJ Hahlen K. Minimal residual disease levels in bone marrow and peripheral blood are comparable in children with T cell acute lymphoblastic leukemia (ALL), but not in precursor-B-ALL. Leukemia (2002) 16(8):1432–6. doi: 10.1038/sj.leu.2402636 12145681

[49] Yegin ZA Can F Gokcen S Sadioglu RE Ozkurt ZN Ilhan C. The impact of pre-transplant cell-free DNA levels on leukemia relapse and transplant-related complications in allogeneic hematopoietic stem cell transplant recipients. Balkan Med J (2020) 37(3):138–43. doi: 10.4274/balkanmedj.galenos.2020.2019.8.25 PMC7161624 31970974

[50] Sehn LH Salles G. Diffuse large b-cell lymphoma. New Engl J Med (2021) 384(9):842–58. doi: 10.1056/NEJMra2027612 PMC8377611 33657296

[51] Swerdlow SH Campo E Pileri SA Harris NL Stein H Siebert R. The 2016 revision of the world health organization classification of lymphoid neoplasms. Blood J Am Soc Hematol (2016) 127(20):2375–90. doi: 10.1182/blood-2016-01-643569 PMC4874220 26980727

[52] Arzuaga-Mendez J Prieto-Fernández E Lopez-Lopez E Martin-Guerrero I García-Ruiz JC García-Orad A. Cell-free DNA as a biomarker in diffuse large b-cell lymphoma: a systematic review. Crit Rev Oncol/Hematol (2019) 139:7–15. doi: 10.1016/j.critrevonc.2019.04.013 31112884

[53] Scherer F Kurtz DM Newman AM Stehr H Craig AF Esfahani MS. Distinct biological subtypes and patterns of genome evolution in lymphoma revealed by circulating tumor DNA. Sci Trans Med (2016) 8(364):364ra155–364ra155. doi: 10.1126/scitranslmed.aai8545 PMC5490494 27831904

[54] Roschewski M Dunleavy K Pittaluga S Moorhead M Pepin F Kong K. Circulating tumour DNA and CT monitoring in patients with untreated diffuse large b-cell lymphoma: a correlative biomarker study. Lancet Oncol (2015) 16(5):541–9. doi: 10.1016/S1470-2045(15)70106-3 PMC4460610 25842160

[55] Ignatiadis M Sledge GW Jeffrey SS. Liquid biopsy enters the clinic–implementation issues and future challenges. Nat Rev Clin Oncol (2021) 18(5):297–312. doi: 10.1038/s41571-020-00457-x 33473219

[56] Huet S Salles G. Potential of circulating tumor DNA for the management of patients with lymphoma. JCO Oncol Practice (2020) 16(9):561–8. doi: 10.1200/JOP.19.00691 32421389

[57] Kurtz DM Green MR Bratman SV Scherer F Liu CL Kunder CA. Noninvasive monitoring of diffuse large b-cell lymphoma by immunoglobulin high-throughput sequencing. Blood (2015) 125(24):3679–87. doi: 10.1182/blood-2015-03-635169 PMC4463733 25887775

[58] Lv L Liu Y. Clinical application of liquid biopsy in non-Hodgkin lymphoma. Front Oncol (2021) 11:658234. doi: 10.3389/fonc.2021.658234 33816315 PMC8013700

[59] Hur JY Kim YJ Yoon SE Son DS Park WY Kim SJ. Plasma cell-free DNA is a prognostic biomarker for survival in patients with aggressive non-Hodgkin lymphomas. Ann Hematol (2020) 99(6):1293–302. doi: 10.1007/s00277-020-04008-3 32296914

[60] Rivas-Delgado A Nadeu F Enjuanes A Casanueva-Eliceiry S Mozas P Magnano L. Mutational landscape and tumor burden assessed by cell-free DNA in diffuse Large b-cell lymphoma in a population-based study. Clin Cancer Res (2021) 27(2):513–21. doi: 10.1158/1078-0432.CCR-20-2558 33122345

[61] Eskandari M Manoochehrabadi S Pashaiefar H Zaimy MA Ahmadvand M. Clinical significance of cell-free DNA as a prognostic biomarker in patients with diffuse large b-cell lymphoma. Blood Res (2019) 54(2):114–9. doi: 10.5045/br.2019.54.2.114 PMC6614089 31309089

[62] Armand P Oki Y Neuberg DS Faham M Cummings C Klinger M. Detection of circulating tumour DNA in patients with aggressive b-cell non-Hodgkin lymphoma. Br J Haematol (2013) 163(1):123–6. doi: 10.1111/bjh.12439 23795711

[63] Kurtz DM Scherer F Jin MC Soo J Craig AF Esfahani MS. Circulating tumor DNA measurements as early outcome predictors in diffuse large b-cell lymphoma. J Clin Oncol (2018) 36(28):2845. doi: 10.1200/JCO.2018.78.5246 30125215 PMC6161832

[64] Rossi D Diop F Spaccarotella E Monti S Zanni M Rasi S. Diffuse large b-cell lymphoma genotyping on the liquid biopsy. Blood J Am Soc Hematol (2017) 129(14):1947–57. doi: 10.1182/blood-2016-05-719641 28096087

[65] Kurtz DM Esfahani MS Scherer F Soo J Jin MC Liu CL. Dynamic risk profiling using serial tumor biomarkers for personalized outcome prediction. Cell (2019) 178(3):699–713.e19. doi: 10.1016/j.cell.2019.06.011 31280963 PMC7380118

[66] Kurtz DM Soo J Co Ting Keh L Alig S Chabon JJ Sworder BJ. Enhanced detection of minimal residual disease by targeted sequencing of phased variants in circulating tumor DNA. Nat Biotechnol (2021) 39(12):1537–47. doi: 10.1038/s41587-021-00981-w PMC8678141 34294911

[67] Meriranta L Alkodsi A Pasanen A Lepistö M Mapar P Blaker YN. Molecular features encoded in the ctDNA reveal heterogeneity and predict outcome in high-risk aggressive b-cell lymphoma. Blood (2022) 139(12):1863–77. doi: 10.1182/blood.2021012852 34932792

[68] Alig S Macaulay CW Kurtz DM Dührsen U Hüttmann A Schmitz C. Short diagnosis-to-treatment interval is associated with higher circulating tumor DNA levels in diffuse large b-cell lymphoma. J Clin Oncol (2021) 39(23):2605–16. doi: 10.1200/JCO.20.02573 PMC8331064 33909455

[69] Shin S-H Kim YJ Lee D Cho D Ko YH Cho J. Analysis of circulating tumor DNA by targeted ultra-deep sequencing across various non-Hodgkin lymphoma subtypes. Leuk Lymphoma (2019) 60(9):2237–46. doi: 10.1080/10428194.2019.1573998 30774000

[70] Hossain NM Dahiya S Le R Abramian AM Kong KA Muffly LS. Circulating tumor DNA assessment in patients with diffuse large b-cell lymphoma following CAR T-cell therapy. Leuk Lymphoma (2019) 60(2):503–6. doi: 10.1080/10428194.2018.1474463 29966461

[71] Di C Jiang Y Li M Juan X Xu C. Circulating exosomal microRNA signature as a noninvasive biomarker for diagnosis of diffuse large b-cell lymphoma. Blood (2018) 132:5406. doi: 10.1182/blood-2018-99-115940

[72] Zare N Haghjooy Javanmard S Mehrzad V Eskandari N Kefayat A. Evaluation of exosomal miR-155, let-7g and let-7i levels as a potential noninvasive biomarker among refractory/relapsed patients, responsive patients and patients receiving r-CHOP. Leuk Lymphoma (2019) 60(8):1877–89. doi: 10.1080/10428194.2018.1563692 30714442

[73] Feng Y Zhong M Zeng S Wang L Liu P Xiao X. Exosome-derived miRNAs as predictive biomarkers for diffuse large b-cell lymphoma chemotherapy resistance. Epigenomics (2019) 11(1):35–51. doi: 10.2217/epi-2018-0123 30211623

[74] Chen Z You L Wang L Huang X Liu H Wei JY. Dual effect of DLBCL-derived EXOs in lymphoma to improve DC vaccine efficacy *in vitro* while favor tumorgenesis in vivo. J Exp Clin Cancer Res (2018) 37(1):190. doi: 10.1186/s13046-018-0863-7 30103789 PMC6090784

[75] Suehara Y Sakata-Yanagimoto M Hattori K Kusakabe M Nanmoku T Sato T. Mutations found in cell-free DNA s of patients with malignant lymphoma at remission can derive from clonal hematopoiesis. Cancer Sci (2019) 110(10):3375–81. doi: 10.1111/cas.14176 PMC6778636 31436356

[76] Jung D Jain P Yao Y Wang M. Advances in the assessment of minimal residual disease in mantle cell lymphoma. J Hematol Oncol (2020) 13(1):127. doi: 10.1186/s13045-020-00961-8 32972438 PMC7513535

[77] Smith MR Jegede O Martin P Till BG Parekh SS Yang DT. ECOG-ACRIN E1411 randomized phase 2 trial of bendamustine-rituximab (BR)-based induction followed by rituximab (R) ± lenalidomide (L) consolidation for mantle cell lymphoma: effect of adding bortezomib to front-line BR induction on PFS. J Clin Oncol (2021) 39(15_suppl):7503. doi: 10.1200/JCO.2021.39.15_suppl.7503

[78] Merryman RW Edwin N Redd R Bsat J Chase M LaCasce A. Rituximab/bendamustine and rituximab/cytarabine induction therapy for transplant-eligible mantle cell lymphoma. Blood Adv (2020) 4(5):858–67. doi: 10.1182/bloodadvances.2019001355 PMC7065472 32126141

[79] Lakhotia R Melani C Dunleavy K Pittaluga S Saba N Lindenberg L. Circulating tumor DNA predicts therapeutic outcome in mantle cell lymphoma. Blood Adv (2022) 6(8):2667–80. doi: 10.1182/bloodadvances.2021006397 PMC9043939 35143622

[80] Kumar A Bantilan KS Jacob AP Park A Schoninger SF Sauter C. Noninvasive monitoring of mantle cell lymphoma by immunoglobulin gene next-generation sequencing in a phase 2 study of sequential chemoradioimmunotherapy followed by autologous stem-cell rescue. Clin Lymphoma Myeloma Leukemia (2021) 21(4):230–7.e12. doi: 10.1016/j.clml.2020.09.007 PMC9476895 33558202

[81] Sarkozy C Huet S Carlton VE Fabiani B Delmer A Jardin F. The prognostic value of clonal heterogeneity and quantitative assessment of plasma circulating clonal IG-VDJ sequences at diagnosis in patients with follicular lymphoma. Oncotarget (2017) 8(5):8765–74. doi: 10.18632/oncotarget.14448 PMC5352439 28060738

[82] Delfau-Larue M-H van der Gucht A Dupuis J Jais J-P Nel I Beldi-Ferchiou A. Total metabolic tumor volume, circulating tumor cells, cell-free DNA: distinct prognostic value in follicular lymphoma. Blood Adv (2018) 2(7):807–16. doi: 10.1182/bloodadvances.2017015164 PMC5894260 29636326

[83] Ubieto AIJ Heredia Y de la Rosa JM Izquierdo AR Rufian L Carrillo J. Minimal residual disease monitoring from liquid biopsy by next generation sequencing in follicular lymphoma patients. Blood (2020) 136:31–3. doi: 10.1182/blood-2020-139256

[84] Galimberti S Luminari S Ciabatti E Grassi S Guerrini F Dondi A. Minimal residual disease after conventional treatment significantly impacts on progression-free survival of patients with follicular lymphoma: the FIL FOLL05 trial. Clin Cancer Res (2014) 20(24):6398–405. doi: 10.1158/1078-0432.CCR-14-0407 25316810

[85] Distler A Lakhotia R Phelan JD Pittaluga S Melani C Muppidi JR. A prospective study of clonal evolution in follicular lymphoma: circulating tumor DNA correlates with overall tumor burden and fluctuates over time without therapy. Blood (2021) 138(Supplement 1):1328. doi: 10.1182/blood-2021-151096

[86] Ladetto M Lobetti-Bodoni C Mantoan B Ceccarelli M Boccomini C Genuardi E. Persistence of minimal residual disease in bone marrow predicts outcome in follicular lymphomas treated with a rituximab-intensive program. Blood J Am Soc Hematol (2013) 122(23):3759–66. doi: 10.1182/blood-2013-06-507319 24085766

[87] Pulsoni A Della Starza I Cappelli LV Tosti ME Annechini G Cavalli M. Minimal residual disease monitoring in early stage follicular lymphoma can predict prognosis and drive treatment with rituximab after radiotherapy. Br J Haematol (2020) 188(2):249–58. doi: 10.1111/bjh.16125 31385309

[88] Luminari S Galimberti S Versari A Tucci A Boccomini C Farina L. Response adapted post induction therapy in follicular lymphoma: updated results of the FOLL12 trial by the fondazione italiana linfomi (FIL). Hematol Oncol (2021) 39. doi: 10.1002/hon.80_2879

[89] Fontanilles M Marguet F Bohers É Viailly PJ Dubois S Bertrand P. Non-invasive detection of somatic mutations using next-generation sequencing in primary central nervous system lymphoma. Oncotarget (2017) 8(29):48157–68. doi: 10.18632/oncotarget.18325 PMC5564634 28636991

[90] Rimelen V Ahle G Pencreach E Zinniger N Debliquis A Zalmaï L. Tumor cell-free DNA detection in CSF for primary CNS lymphoma diagnosis. Acta Neuropathol Commun (2019) 7(1):43. doi: 10.1186/s40478-019-0692-8 30885253 PMC6421652

[91] Hiemcke-Jiwa LS Leguit RJ Snijders TJ Bromberg JEC Nierkens S Jiwa NM. MYD88 p.(L265P) detection on cell-free DNA in liquid biopsies of patients with primary central nervous system lymphoma. Br J Haematol (2019) 185(5):974–7. doi: 10.1111/bjh.15674 30408153

[92] Yamagishi Y Sasaki N Nakano Y Matushita Y Omura T Shimizu S. Liquid biopsy of cerebrospinal fluid for MYD88 L265P mutation is useful for diagnosis of central nervous system lymphoma. Cancer Sci (2021) 112(11):4702–10. doi: 10.1111/cas.15133 PMC8586690 34523186

[93] Watanabe J Natsumeda M Okada M Kobayashi D Kanemaru Y Tsukamoto Y. High detection rate of MYD88 mutations in cerebrospinal fluid from patients with CNS lymphomas. JCO Precis Oncol (2019) 3):1–13. doi: 10.1200/PO.18.00308 35100686

[94] Bobillo S Crespo M Escudero L Mayor R Raheja P Carpio C. Cell free circulating tumor DNA in cerebrospinal fluid detects and monitors central nervous system involvement of b-cell lymphomas. Haematologica (2021) 106(2):513–21. doi: 10.3324/haematol.2019.241208 PMC7849551 32079701

[95] Heger J-M Mattlener J Gödel P Balke-Want H Sieg N Kutsch N. Noninvasive, dynamic risk profiling of primary central nervous system lymphoma by peripheral blood ctdna-sequencing. Blood (2022) 140(Supplement 1):3537–8. doi: 10.1182/blood-2022-162420

[96] Mutter JA Alig S Lauer EM Esfahani MS Mitschke J Kurtz DM. Profiling of circulating tumor DNA for noninvasive disease detection, risk stratification, and MRD monitoring in patients with CNS lymphoma. Blood (2021) 138(Supplement 1):6. doi: 10.1182/blood-2021-149644 34236427

[97] Cook LB Phillips AA. How I treat adult T-cell leukemia/lymphoma. Blood (2021) 137(4):459–70. doi: 10.1182/blood.2019004045 33075812

[98] Hayashida M Maekawa F Chagi Y Iioka F Kobashi Y Watanabe M. Combination of multicolor flow cytometry for circulating lymphoma cells and tests for the RHOA(G17V) and IDH2(R172) hot-spot mutations in plasma cell-free DNA as liquid biopsy for the diagnosis of angioimmunoblastic T-cell lymphoma. Leuk Lymphoma (2020) 61(10):2389–98. doi: 10.1080/10428194.2020.1768382 32476550

[99] Miljkovic MD Melani C Pittaluga S Lakhotia R Lucas N Jacob A. Next-generation sequencing-based monitoring of circulating tumor DNA reveals clonotypic heterogeneity in untreated PTCL. Blood Adv (2021) 5(20):4198–210. doi: 10.1182/bloodadvances.2020003679 PMC8945631 34432874

[100] Casasnovas RO Bouabdallah R Brice P Lazarovici J Ghesquieres H Stamatoullas A. PET-adapted treatment for newly diagnosed advanced Hodgkin lymphoma (AHL2011): a randomised, multicentre, non-inferiority, phase 3 study. Lancet Oncol (2019) 20(2):202–15. doi: 10.1016/S1470-2045(18)30784-8 30658935

[101] Camus V Jardin F. Cell-free DNA for the management of classical Hodgkin lymphoma. Pharm (Basel) (2021) 14(3). doi: 10.3390/ph14030207 PMC7998645 33801462

[102] Bessi L Viailly PJ Bohers E Ruminy P Maingonnat C Bertrand P. Somatic mutations of cell-free circulating DNA detected by targeted next-generation sequencing and digital droplet PCR in classical Hodgkin lymphoma. Leuk Lymphoma (2019) 60(2):498–502. doi: 10.1080/10428194.2018.1492123 30068243

[103] Schmitz R Stanelle J Hansmann ML Küppers R. Pathogenesis of classical and lymphocyte-predominant Hodgkin lymphoma. Annu Rev Pathol (2009) 4:151–74. doi: 10.1146/annurev.pathol.4.110807.092209 19400691

[104] Stewart CM Kothari PD Mouliere F Mair R Somnay S Benayed R. The value of cell-free DNA for molecular pathology. J Pathol (2018) 244(5):616–27. doi: 10.1002/path.5048 PMC6656375 29380875

[105] Spina V Bruscaggin A Cuccaro A Martini M Di Trani M Forestieri G. Circulating tumor DNA reveals genetics, clonal evolution, and residual disease in classical Hodgkin lymphoma. Blood (2018) 131(22):2413–25. doi: 10.1182/blood-2017-11-812073 29449275

[106] Desch AK Hartung K Botzen A Brobeil A Rummel M Kurch L. Genotyping circulating tumor DNA of pediatric Hodgkin lymphoma. Leukemia (2020) 34(1):151–66. doi: 10.1038/s41375-019-0541-6 31431735

[107] Camus V Stamatoullas A Mareschal S Viailly PJ Sarafan-Vasseur N Bohers E. Detection and prognostic value of recurrent exportin 1 mutations in tumor and cell-free circulating DNA of patients with classical Hodgkin lymphoma. Haematologica (2016) 101(9):1094–101. doi: 10.3324/haematol.2016.145102 PMC5060026 27479820

[108] Yeh P Hunter T Sinha D Ftouni S Wallach E Jiang D. Circulating tumour DNA reflects treatment response and clonal evolution in chronic lymphocytic leukaemia. Nat Commun (2017) 8:14756. doi: 10.1038/ncomms14756 28303898 PMC5357854

[109] Rasche L Chavan SS Stephens OW Patel PH Tytarenko R Ashby C. Spatial genomic heterogeneity in multiple myeloma revealed by multi-region sequencing. Nat Commun (2017) 8(1):268. doi: 10.1038/s41467-017-00296-y 28814763 PMC5559527

[110] Levin A Hari P Dhakal B. Novel biomarkers in multiple myeloma. Transl Res (2018) 201:49–59. doi: 10.1016/j.trsl.2018.05.003 30301522

[111] Rawstron AC Orfao A Beksac M Bezdickova L Brooimans R Bumbea H. Report of the European myeloma network on multiparametric flow cytometry in multiple myeloma and related disorders. Haematologica (2008) 93(3):431–8. doi: 10.3324/haematol.11080 18268286

[112] Flores-Montero J Sanoja-Flores L Paiva B Puig N García-Sánchez O Böttcher S. Next generation flow for highly sensitive and standardized detection of minimal residual disease in multiple myeloma. Leukemia (2017) 31(10):2094–103. doi: 10.1038/leu.2017.29 PMC5629369 28104919

[113] Martinez-Lopez J Lahuerta JJ Pepin F González M Barrio S Ayala R. Prognostic value of deep sequencing method for minimal residual disease detection in multiple myeloma. Blood J Am Soc Hematol (2014) 123(20):3073–9. doi: 10.1182/blood-2014-01-550020 PMC4023416 24646471

[114] Ching T Duncan ME Newman-Eerkes T McWhorter MM Tracy JM Steen MS. Analytical evaluation of the clonoSEQ assay for establishing measurable (minimal) residual disease in acute lymphoblastic leukemia, chronic lymphocytic leukemia, and multiple myeloma. BMC Cancer (2020) 20(1):1–15. doi: 10.1186/s12885-020-07077-9 PMC7325652 32605647

[115] Walzer S Krenberger S Vollmer L Hewitt T Eckert B. A cost impact analysis of clonoSEQ^®^ as a valid and CE-certified minimal residual disease (MRD) diagnostic compared to no MRD testing in multiple myeloma in Germany. Oncol Ther (2021) 9:607–19. doi: 10.1007/s40487-021-00169-x PMC8593124 34480748

[116] Oliva S Genuardi E Belotti A Frascione PMM Galli M Capra A. Minimal residual disease evaluation by multiparameter flow cytometry and next generation sequencing in the forte trial for newly diagnosed multiple myeloma patients. Blood (2019) 134:4322. doi: 10.1182/blood-2019-124645

[117] Avet-Loiseau H Bene MC Wuilleme S Corre J Attal M Arnulf B. Concordance of post-consolidation minimal residual disease rates by multiparametric flow cytometry and next-generation sequencing in CASSIOPEIA. Clin Lymphoma Myeloma Leukemia (2019) 19(10):e3–4. doi: 10.1016/j.clml.2019.09.005

[118] Hussaini MO Srivastava J Lee LW Nishihori T Shah B Alsina M. Moffitt Cancer center 2-year single-institution experience with next-generation sequencing minimal residual disease detection: clinical utility, application, and correlation with outcomes in plasma cell and lymphoid malignancies. Blood (2019) 134:4654. doi: 10.1182/blood-2019-129846

[119] Landgren O Devlin S Boulad M Mailankody S. Role of MRD status in relation to clinical outcomes in newly diagnosed multiple myeloma patients: a meta-analysis. Bone Marrow Transpl (2016) 51(12):1565–8. doi: 10.1038/bmt.2016.222 PMC5571752 27595280

[120] Munshi NC Avet-Loiseau H Rawstron AC Owen RG Child JA Thakurta A. Association of minimal residual disease with superior survival outcomes in patients with multiple myeloma: a meta-analysis. JAMA Oncol (2017) 3(1):28–35. doi: 10.1001/jamaoncol.2016.3160 27632282 PMC5943640

[121] Munshi NC Avet-Loiseau H Anderson KC Neri P Paiva B Samur M. A large meta-analysis establishes the role of MRD negativity in long-term survival outcomes in patients with multiple myeloma. Blood Adv (2020) 4(23):5988–99. doi: 10.1182/bloodadvances.2020002827 PMC7724898 33284948

[122] Costa LJ Derman BA Bal S Sidana S Chhabra S Silbermann R. International harmonization in performing and reporting minimal residual disease assessment in multiple myeloma trials. Leukemia (2021) 35(1):18–30. doi: 10.1038/s41375-020-01012-4 32778736

[123] Anderson K Auclair D Adam SJ Agarwal A Anderson M Avet-Loiseau H. Minimal residual disease in myeloma: application for clinical care and new drug registration. Clin Cancer Res (2021) 27(19):5195–12. doi: 10.1158/1078-0432.CCR-21-1059 PMC9662886 34321279

[124] Gormley NJ Farrell AT Pazdur R. Minimal residual disease as a potential surrogate end point–lingering questions. JAMA Oncol (2017) 3(1):18–20. doi: 10.1001/jamaoncol.2016.3112 27632052

[125] Anderson KC Auclair D Kelloff GJ Sigman CC Avet-Loiseau H Farrell AT. The role of minimal residual disease testing in myeloma treatment selection and drug development: current value and future ApplicationsMinimal residual disease in myeloma. Clin Cancer Res (2017) 23(15):3980–93. doi: 10.1158/1078-0432.CCR-16-2895 28428191

[126] Holstein SA Al-Kadhimi Z Costa LJ Hahn T Hari P Hillengass J. Summary of the third annual blood and marrow transplant clinical trials network myeloma intergroup workshop on minimal residual disease and immune profiling. Biol Blood Marrow Transpl (2020) 26(1):e7–e15. doi: 10.1016/j.bbmt.2019.09.015 PMC6942175 31526843

[127] Zhang L Beasley S Prigozhina NL Higgins R Ikeda S Lee FY. Detection and characterization of circulating tumour cells in multiple myeloma. J Circ biomark (2016) 5:10. doi: 10.5772/64124 28936258 PMC5548310

[128] Sata H Shibayama H Maeda I Habuchi Y Nakatani E Fukushima K. Quantitative polymerase chain reaction analysis with allele-specific oligonucleotide primers for individual IgH VDJ regions to evaluate tumor burden in myeloma patients. Exp Hematol (2015) 43(5):374–81.e2. doi: 10.1016/j.exphem.2015.01.002 25591497

[129] Vasco-Mogorrón MA Campillo JA Periago A Cabañas V Berenguer M García-Garay MC. Blood-based risk stratification for pre-malignant and symptomatic plasma cell neoplasms to improve patient management. Am J Cancer Res (2021) 11(6):2736–53. PMC8263680 34249425

[130] Bianchi G Richardson PG Anderson KC. Promising therapies in multiple myeloma. Blood (2015) 126(3):300–10. doi: 10.1182/blood-2015-03-575365 PMC4560339 26031917

[131] Garcés JJ Cedena MT Puig N Burgos L Perez JJ Cordon L. Circulating tumor cells for the staging of patients with newly diagnosed transplant-eligible multiple myeloma. J Clin Oncol (2022) 40(27):3151–61. doi: 10.1200/JCO.21.01365 35666958

[132] Oberle A Brandt A Voigtlaender M Thiele B Radloff J Schulenkorf A. Monitoring multiple myeloma by next-generation sequencing of V(D)J rearrangements from circulating myeloma cells and cell-free myeloma DNA. Haematologica (2017) 102(6):1105–11. doi: 10.3324/haematol.2016.161414 PMC5451343 28183851

[133] Long X Xu Q Lou Y Li C Gu J Cai H. The utility of non-invasive liquid biopsy for mutational analysis and minimal residual disease assessment in extramedullary multiple myeloma. Br J Haematol (2020) 189(2):e45–e8. doi: 10.1111/bjh.16440 32191818

[134] Deshpande S Tytarenko RG Wang Y Boyle EM Ashby C Schinke CD. Monitoring treatment response and disease progression in myeloma with circulating cell-free DNA. Eur J Haematol (2021) 106(2):230–40. doi: 10.1111/ejh.13541 33107092

[135] Yasui H Kobayashi M Sato K Kondoh K Ishida T Kaito Y. Circulating cell-free DNA in the peripheral blood plasma of patients is an informative biomarker for multiple myeloma relapse. Int J Clin Oncol (2021) 26(11):2142–50. doi: 10.1007/s10147-021-01991-z 34259983

[136] Rustad EH Coward E Skytøen ER Misund K Holien T Standal T. Monitoring multiple myeloma by quantification of recurrent mutations in serum. Haematologica (2017) 102(7):1266–72. doi: 10.3324/haematol.2016.160564 PMC5566041 28385781

[137] Pugh TJ. Circulating tumour DNA for detecting minimal residual disease in multiple myeloma. Semin Hematol (2018) 55(1):38–40. doi: 10.1053/j.seminhematol.2018.03.002 29759151

[138] Manier S Park J Capelletti M Bustoros M Freeman S Ha G. Whole-exome sequencing of cell-free DNA and circulating tumor cells in multiple myeloma. Nat Commun (2018) 9(1):1–11. doi: 10.1038/s41467-018-04001-5 29703982 PMC5923255

[139] Ntanasis-Stathopoulos I Gavriatopoulou M Terpos E Fotiou D Kastritis E Dimopoulos MA. Monitoring plasma cell dyscrasias with cell-free DNA analysis. Clin Lymphoma Myeloma Leukemia (2020) 20(11):e905–e9. doi: 10.1016/j.clml.2020.06.025 32723621

[140] Mithraprabhu S Khong T Ramachandran M Chow A Klarica D Mai L. Circulating tumour DNA analysis demonstrates spatial mutational heterogeneity that coincides with disease relapse in myeloma. Leukemia (2017) 31(8):1695–705. doi: 10.1038/leu.2016.366 27899805

[141] Kis O Kaedbey R Chow S Danesh A Dowar M Li T. Circulating tumour DNA sequence analysis as an alternative to multiple myeloma bone marrow aspirates. Nat Commun (2017) 8(1):15086. doi: 10.1038/ncomms15086 28492226 PMC5437268

[142] Gerber B Manzoni M Spina V Bruscaggin A Lionetti M Fabris S. Circulating tumor DNA as a liquid biopsy in plasma cell dyscrasias. Haematologica (2018) 103(6):e245. doi: 10.3324/haematol.2017.184358 29472358 PMC6058782

[143] Treon SP Xu L Guerrera ML Jimenez C Hunter ZR Liu X. Genomic landscape of waldenström macroglobulinemia and its impact on treatment strategies. J Clin Oncol (2020) 38(11):1198–208. doi: 10.1200/JCO.19.02314 PMC7351339 32083995

[144] Bagratuni T Ntanasis-Stathopoulos I Gavriatopoulou M Mavrianou-Koutsoukou N Liacos C Patseas D. Detection of MYD88 and CXCR4 mutations in cell-free DNA of patients with IgM monoclonal gammopathies. Leukemia (2018) 32(12):2617–25. doi: 10.1038/s41375-018-0197-7 PMC6286389 30026568

[145] Ntanasis-Stathopoulos I Bagratuni T Gavriatopoulou M Patseas D Liacos C Kanellias N. Cell-free DNA analysis for the detection of MYD88 and CXCR4 mutations in IgM monoclonal gammopathies; an update with clinicopathological correlations. Am J Hematol (2020) 95(6):E148–e50. doi: 10.1002/ajh.25802 32242972

[146] Drandi D Genuardi E Dogliotti I Ferrante M Jiménez C Guerrini F. Highly sensitive MYD88(L265P) mutation detection by droplet digital polymerase chain reaction in waldenström macroglobulinemia. Haematologica (2018) 103(6):1029–37. doi: 10.3324/haematol.2017.186528 PMC6058774 29567768

[147] Hiemcke-Jiwa LS Minnema MC Radersma-van Loon JH Jiwa NM de Boer M Leguit RJ. The use of droplet digital PCR in liquid biopsies: a highly sensitive technique for MYD88 p. (L265P) Detection Cerebrospinal Fluid Hematol Oncol (2018) 36(2):429–35. doi: 10.1002/hon.2489 29210102

[148] Cross N White H Colomer D Ehrencrona H Foroni L Gottardi E. Laboratory recommendations for scoring deep molecular responses following treatment for chronic myeloid leukemia. Leukemia (2015) 29(5):999–1003. doi: 10.1038/leu.2015.29 25652737 PMC4430701

[149] Hochhaus A Baccarani M Silver RT Schiffer C Apperley JF Cervantes F. European LeukemiaNet 2020 recommendations for treating chronic myeloid leukemia. Leukemia (2020) 34(4):966–84. doi: 10.1038/s41375-020-0776-2 PMC7214240 32127639

[150] Mori S Vagge E Le Coutre P Abruzzese E Martino B Pungolino E. Age and d PCR can predict relapse in CML patients who discontinued imatinib: the ISAV study. Am J Hematol (2015) 90(10):910–4. doi: 10.1002/ajh.24120 26178642

[151] Berdeja JG Heinrich MC Dakhil SR Goldberg SL Wadleigh M Kuriakose P. Rates of deep molecular response by digital and conventional PCR with frontline nilotinib in newly diagnosed chronic myeloid leukemia: a landmark analysis. Leuk Lymphoma (2019) 60(10):2384–93. doi: 10.1080/10428194.2019.1590569 30912699

[152] Goh H-G Lin M Fukushima T Saglio G Kim D Choi S-Y. Sensitive quantitation of minimal residual disease in chronic myeloid leukemia using nanofluidic digital polymerase chain reaction assay. Leuk Lymphoma (2011) 52(5):896–904. doi: 10.3109/10428194.2011.555569 21338281

[153] Wang WJ Zheng CF Liu Z Tan YH Chen XH Zhao BL. Droplet digital PCR for BCR/ABL (P210) detection of chronic myeloid leukemia: a high sensitive method of the minimal residual disease and disease progression. Eur J Haematol (2018) 101(3):291–6. doi: 10.1111/ejh.13084 29691899

[154] Rovida E Marzi I Cipolleschi MG Sbarba PD. One more stem cell niche: how the sensitivity of chronic myeloid leukemia cells to imatinib mesylate is modulated within a “hypoxic” environment. Hypoxia (2014) 2:1. doi: 10.2147/hp.s51812 27774462 PMC5045050

[155] Krumbholz M Karl M Tauer JT Thiede C Rascher W Suttorp M. Genomic BCR-ABL1 breakpoints in pediatric chronic myeloid leukemia. Genes Chromosomes Cancer (2012) 51(11):1045–53. doi: 10.1002/gcc.21989 22887688

[156] Lund HL Hughesman CB McNeil K Clemens S Hocken K Pettersson R. Initial diagnosis of chronic myelogenous leukemia based on quantification of m-BCR status using droplet digital PCR. Anal Bioanal Chem (2016) 408:1079–94. doi: 10.1007/s00216-015-9204-2 26631023

[157] Cumbo C Impera L Minervini CF Orsini P Anelli L Zagaria A. Genomic BCR-ABL1 breakpoint characterization by a multi-strategy approach for “personalized monitoring” of residual disease in chronic myeloid leukemia patients. Oncotarget (2018) 9(13):10978. doi: 10.18632/oncotarget.23971 29541390 PMC5834283

[158] Bernardi S Foroni C Zanaglio C Re F Polverelli N Turra A. Feasibility of tumor−derived exosome enrichment in the onco−hematology leukemic model of chronic myeloid leukemia. Int J Mol Med (2019) 44(6):2133–44. doi: 10.3892/ijmm.2019.4372 PMC6844640 31638195

[159] Polverelli SBMMN Russo D. Exosomes in chronic myeloid leukemia: are we reading a new reliable message? Acta Haematol (2020) 143:509–10. doi: 10.1159/000505088 31922494

[160] Bernardi S Malagola M Zanaglio C Polverelli N Dereli Eke E D’Adda M. Digital PCR improves the quantitation of DMR and the selection of CML candidates to TKIs discontinuation. Cancer Med (2019) 8(5):2041–55. doi: 10.1002/cam4.2087 PMC6536984 30950237

[161] Zanaglio C Bernardi S Gandolfi L Farina M Re F Polverelli N. RT-qPCR versus digital PCR: how do they impact differently on clinical management of chronic myeloid leukemia patients? Case Rep Oncol (2020) 13(3):1263–9. doi: 10.1159/000510440 PMC7670369 33250741

[162] Kjaer L Skov V Andersen MT Aggerholm A Clair P Gniot M. Variant-specific discrepancy when quantitating BCR-ABL1 e13a2 and e14a2 transcripts using the Europe against cancer qPCR assay. Eur J Haematol (2019) 103(1):26–34. doi: 10.1111/ejh.13238 30985947

[163] Dueck ME Lin R Zayac A Gallagher S Chao AK Jiang L. Precision cancer monitoring using a novel, fully integrated, microfluidic array partitioning digital PCR platform. Sci Rep (2019) 9(1):1–9. doi: 10.1038/s41598-019-55872-7 31862911 PMC6925289

[164] Chung H-J Hur M Yoon S Hwang K Lim H-S Kim H. Performance evaluation of the QXDx BCR-ABL% IS droplet digital PCR assay. Ann Lab Med (2020) 40(1):72–5. doi: 10.3343/alm.2020.40.1.72 PMC6713652 31432643

[165] Fava C Bernardi S Gottardi EM Lorenzatti R Galeotti L Ceccherini F. Alignment of Qx100/Qx200 droplet digital (Bio-rad) and QuantStudio 3D (Thermofisher) digital PCR for quantification of BCR-ABL1 in ph+ chronic myeloid leukemia. Diseases (2021) 9(2):35. 34062996 10.3390/diseases9020035 PMC8161814

[166] Maier J Lange T Cross M Wildenberger K Niederwieser D Franke G-N. Optimized digital droplet PCR for BCR-ABL. J Mol Diagnostics (2019) 21(1):27–37. doi: 10.1016/j.jmoldx.2018.08.012 30347270

[167] Herrmann H Sadovnik I Cerny-Reiterer S Rülicke T Stefanzl G Willmann M. (CD26) defines leukemic stem cells (LSC) in chronic myeloid leukemia. Blood J Am Soc Hematol (2014) 123(25):3951–62. doi: 10.1182/blood-2013-10-536078 24778155

[168] Bocchia M Sicuranza A Abruzzese E Iurlo A Sirianni S Gozzini A. Residual peripheral blood CD26+ leukemic stem cells in chronic myeloid leukemia patients during TKI therapy and during treatment-free remission. Front Oncol (2018) 8:194. doi: 10.3389/fonc.2018.00194 29900128 PMC5988870

[169] Cumbo C Anelli L Specchia G Albano F. Monitoring of minimal residual disease (MRD) in chronic myeloid leukemia: recent advances. Cancer Manag Res (2020) 12:3175. doi: 10.2147/CMAR.S232752 32440215 PMC7211966

[170] Walter W Pohlkamp C Meggendorfer M Nadarajah N Kern W Haferlach C. Artificial intelligence in hematological diagnostics: game changer or gadget? Blood Rev (2022), 101019. doi: 10.1016/j.blre.2022.101019 36241586

[171] FDA. Artificial intelligence and machine learning (AI/ML)-enabled medical devices (2022). Available at: https://www.fda.gov/medical-devices/software-medical-device-samd/artificial-intelligence-and-machine-learning-aiml-enabled-medical-devices.

[172] Meskó B Görög M. A short guide for medical professionals in the era of artificial intelligence. NPJ Digit Med (2020) 3:126. doi: 10.1038/s41746-020-00333-z 33043150 PMC7518439

[173] Kimura K Tabe Y Ai T Takehara I Fukuda H Takahashi H. A novel automated image analysis system using deep convolutional neural networks can assist to differentiate MDS and AA. Sci Rep (2019) 9(1):13385. doi: 10.1038/s41598-019-49942-z 31527646 PMC6746738

[174] Eckardt JN Schmittmann T Riechert S Kramer M Sulaiman AS Sockel K. Deep learning identifies acute promyelocytic leukemia in bone marrow smears. BMC Cancer (2022) 22(1):201. doi: 10.1186/s12885-022-09307-8 35193533 PMC8864866

[175] Sasaki K Jabbour EJ Ravandi F Konopleva M Borthakur G Wierda WG. The LEukemia artificial intelligence program (LEAP) in chronic myeloid leukemia in chronic phase: a model to improve patient outcomes. Am J Hematol (2021) 96(2):241–50. doi: 10.1002/ajh.26047 PMC9022629 33180322

[176] Ko BS Wang YF Li JL Li CC Weng PF Hsu SC. Clinically validated machine learning algorithm for detecting residual diseases with multicolor flow cytometry analysis in acute myeloid leukemia and myelodysplastic syndrome. EBioMedicine (2018) 37:91–100. doi: 10.1016/j.ebiom.2018.10.042 30361063 PMC6284584

[177] Reiter M Diem M Schumich A Maurer-Granofszky M Karawajew L Rossi JG. Automated flow cytometric MRD assessment in childhood acute b- lymphoblastic leukemia using supervised machine learning. Cytometry A (2019) 95(9):966–75. doi: 10.1002/cyto.a.23852 31282025

[178] Salama ME Otteson GE Camp JJ Seheult JN Jevremovic D Holmes DR 3rd. Artificial intelligence enhances diagnostic flow cytometry workflow in the detection of minimal residual disease of chronic lymphocytic leukemia. Cancers (Basel) (2022) 14(10). doi: 10.3390/cancers14102537 PMC9139233 35626140

[179] Hoffmann J Thrun MC Röhnert MA von Bonin M Oelschlägel U Neubauer A. Identification of critical hemodilution by artificial intelligence in bone marrow assessed for minimal residual disease analysis in acute myeloid leukemia: the Cinderella method. Cytometry A (2022). doi: 10.1002/cyto.a.24686 36030398

[180] Guerrero C Puig N Cedena MT Goicoechea I Perez C Garcés JJ. A machine learning model based on tumor and immune biomarkers to predict undetectable MRD and survival outcomes in multiple myeloma. Clin Cancer Res (2022) 28(12):2598–609. doi: 10.1158/1078-0432.CCR-21-3430 35063966

[181] van der Velden BHM Kuijf HJ Gilhuijs KGA Viergever MA. Explainable artificial intelligence (XAI) in deep learning-based medical image analysis. Med Image Analysis (2022) 79:102470. doi: 10.1016/j.media.2022.102470 35576821

[182] Topol EJ. High-performance medicine: the convergence of human and artificial intelligence. Nat Med (2019) 25(1):44–56. doi: 10.1038/s41591-018-0300-7 30617339

